# PPTC7 acts as an essential co-factor of the SCF^FBXL4^ ubiquitin ligase complex to restrict BNIP3/3L-dependent mitophagy

**DOI:** 10.1038/s41419-025-07463-w

**Published:** 2025-03-01

**Authors:** Xiayun Xu, Yingji Chen, Siqi Fei, Xinyue Jiang, Xiaoting Zhou, Yimeng Xue, Yao Li, Shi-Min Zhao, Yan Huang, Chenji Wang

**Affiliations:** 1 State Key Laboratory of Genetic Engineering, Shanghai Stomatological Hospital & School of Stomatology, MOE Engineering Research Center of Gene Technology, Shanghai Engineering Research Center of Industrial Microorganisms, School of Life Sciences, Fudan University, Shanghai, China; 2 Obstetrics & Gynecology Hospital of Fudan University, Shanghai Key Laboratory of Metabolic Remodeling and Health, State Key Laboratory of Genetic Engineering, School of Life Sciences, Children’s Hospital of Fudan University, and Institutes of Biomedical Sciences, Fudan University, Shanghai, China

**Keywords:** Proteasome, Mitophagy

## Abstract

Mitophagy is a selective process that targets the damaged, dysfunctional, or superfluous mitochondria for degradation through autophagy. The SCF^FBXL4^ E3 ubiquitin ligase complex suppresses basal mitophagy by targeting BNIP3 and BNIP3L, two key mitophagy cargo receptors, for ubiquitin-proteasomal degradation. *FBXL4* loss-of-function mutations lead to excessive BNIP3/3L-dependent mitophagy, thereby causing a devastating multi-system disorder called mitochondrial DNA depletion syndrome, type 13 (MTDPS13). PPTC7, a mitochondrial matrix phosphatase, is essential for proper mitochondrial function and biogenesis. Here, we show that a proportion of PPTC7 is located on the outer mitochondrial membrane, where it interacts with FBXL4 and BNIP3/3L. PPTC7 decreases BNIP3/3L protein stability in a protein phosphatase activity-independent manner. Using in vitro cell culture and *Pptc7* knockout mouse model, we demonstrate that PPTC7 deficiency activates high levels of basal mitophagy in a BNIP3/3L-dependent manner. Mechanistically, PPTC7 facilitates SCF^FBXL4^-mediated ubiquitin-proteasomal degradation of BNIP3/3L. Overall, these findings establish PPTC7 as an essential co-factor of the SCF^FBXL4^ complex and a suppressor of BNIP3/3L-dependent mitophagy.

## Introduction

Mitochondria are essential organelles in eukaryotic cells, playing vital roles in crucial cellular processes such as bioenergetics, metabolism, and signal transduction. Mitophagy is a cellular process involving the targeted degradation and removal of damaged, dysfunctional, or superfluous mitochondria by autophagy, which is essential for maintaining cellular homeostasis and mitochondrial quality control. This selective removal of impaired mitochondria helps to prevent the accumulation of defective organelles, thereby reducing oxidative stress, maintaining energy production balance, and promoting cellular homeostasis [1–3]. Dysregulation of mitophagy has been linked to a wide range of conditions, including neurodegenerative disorders, metabolic disorders, as well as cancer and cardiovascular diseases [1–3]. Impaired mitophagy can lead to the buildup of damaged mitochondria, resulting in increased oxidative damage, inflammation, energy deficits, and ultimately contributing to disease progression and pathology [2, 4]. Conversely, excessive mitophagy under certain pathological conditions can decrease mitochondrial content, burdening the remaining organelles and ultimately triggering mitophagic cell death [5, 6].

Most studies on mitophagy have focused on a canonical pathway involving Parkinson’s disease-related proteins PINK1 and Parkin, particularly their roles in depolarization-induced mitophagy in vitro [7, 8]. However, recent research using Mito-QC reporter in mice and Drosophila models revealed that basal mitophagy activity is largely independent of the PINK1/Parkin pathway [9]. Alternatively, mitochondrial outer membrane receptors, including but not limited to BNIP3, BNIP3L/NIX, FUNDC1, and BCL2L13, also play crucial roles in mitophagy [4, 10]. These receptors serve as bridges between mitochondria marked for degradation and the autophagic machinery, ensuring efficient removal of these organelles. By interacting with ATG8 family proteins (LC3/GABARAPs) on the autophagosomal membrane, these receptors help to trigger the formation of autophagosomes around the targeted mitochondria, leading to their subsequent degradation [11]. Among them, BNIP3 and BNIP3L are transcriptionally activated by the transcription factor hypoxia-inducible factor (HIF1α) under hypoxia, thereby promoting cellular adaptation to low-oxygen conditions and ensuring efficient metabolic remodeling for cell survival [12].

We and others have recently demonstrated that the ubiquitin-proteasomal degradation of BNIP3 and BNIP3L is tightly regulated by the SCF^FBXL4^ E3 ubiquitin ligase complex [13–16]. FBXL4, a member of the F-box protein family, serves as a receptor for substrates to facilitate their recognition by the Skp1-Cul1-F-box (SCF) E3 ubiquitin ligase complex. Importantly, biallelic mutation in the *FBXL4* gene leads to Encephalomyopathic mitochondrial DNA (mtDNA) depletion syndrome 13 (MTDPS13), a severe infantile-onset genetic disorder characterized by excessive mitophagy in patient tissues and organs [17–19]. MTDPS13-associated FBXL4 mutations disrupt the assembly of an active SCF^FBXL4^ complex, resulting in the robust accumulation of BNIP3/3L proteins. This triggers high levels of mitophagy even under basal conditions, underscoring the harmful effects of excessive mitophagy on cellular homeostasis [13–16].

Although the pathophysiological roles of the SCF^FBXL4^ complex in mitophagy have been clearly established, little is known about how this complex is regulated. Here, we demonstrate that PPTC7, a mitochondrial matrix phosphatase [20], interacts with FBXL4 and BNIP3/3L on the mitochondrial outer membrane. PPTC7 deficiency leads to excessive mitophagy in a BNIP3/3L-dependent manner but independent of its protein phosphatase activity. We establish PPTC7 as an essential co-factor for the SCF^FBXL4^ E3 complex to facilitate ubiquitination and degradation of BNIP3/3L.

## Results

### Identification of BNIP3/3L and FBXL4 as PPTC7-interacting proteins

**Fig. 1:**
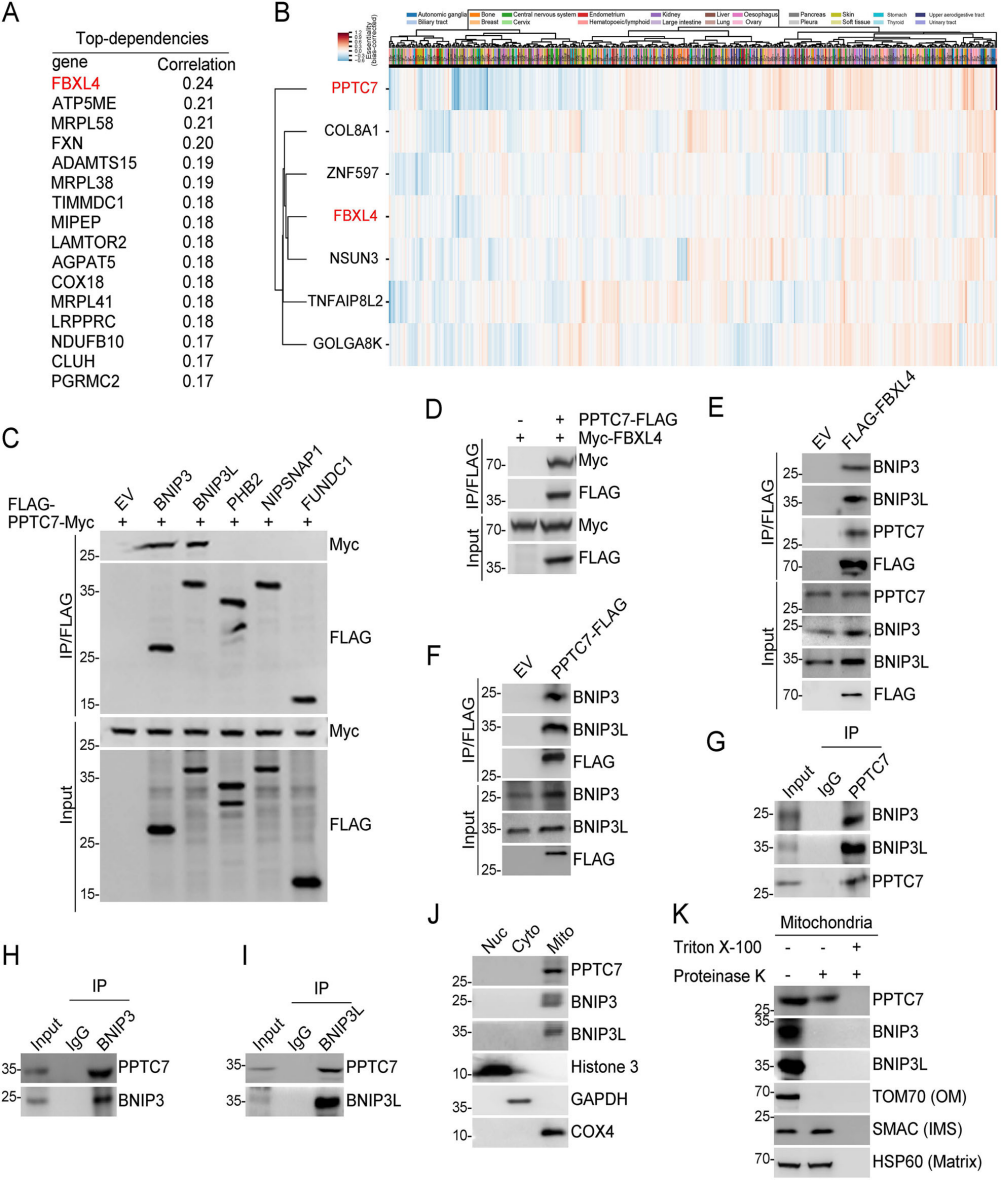
Identification of BNIP3/3L and FBXL4 as PPTC7 interacting proteins. **A** The top 16 genes co-dependent on PPTC7 were identified from a CRISPR screen dataset comprising 990 cell lines obtained from the Achilles DepMap project. Co-dependency values are represented by correlation coefficients. **B** A co-essential module containing PPTC7, FBXL4, and associated proteins was identified from a published gene co-dependency dataset (https://mitra.stanford.edu/bassik/michael/cluster_heatmaps/). The heatmap illustrates the clustering of genes based on their co-dependency profiles across different cancer types. **C**–**F** Western blot (WB) analysis of the indicated proteins in the input and co-immunoprecipitation (co-IP) samples of anti-FLAG antibody obtained from 293 T cells transfected with indicated plasmids. **G**–**I** WB analysis of the input and co-IP samples of from 293 T cells immunoprecipitated with IgG, PPTC7(**G**), BNIP3(**H**), and BNIP3L(**I**) antibody. **J** Subcellular localization of PPTC7, BNIP3, and BNIP3L was determined in HeLa cells. Cytoplasmic, nuclear, and mitochondrial fractions were prepared, and protein distribution was assessed by WB. Histone 3, GAPDH, and COX4 served as markers for nuclear, cytoplasmic, and mitochondrial compartments, respectively. **K** Proteinase K protection assays were performed to assess the localization of PPTC7, BNIP3, and BNIP3L within mitochondria. Mitochondria were isolated from HeLa cells, treated with proteinase K in the presence or absence of Triton X-100 (to disrupt the outer membrane), and analyzed by WB. TOM70 (outer membrane, OM), SMAC (intermembrane space, IMS), and HSP60 (matrix) served as controls to confirm sub-mitochondrial localization.

To elucidate the unidentified functional partners of FBXL4, we analyzed the genetic co-dependency between FBXL4 and other proteins using Broad’s 21Q2 DepMap dataset [21]. This dataset, derived from large-scale loss-of-function sgRNA screens for vulnerabilities in 990 cancer cell lines, allows the identification of genes with similar functions or pathways [22, 23]. Among the top correlated genes, FBXL4 showed the strongest positive correlation with PPTC7 (Fig. 1A). Notably, FBXL4 and PPTC7 co-existed within a co-essential module based on the genetic co-dependency dataset (Fig. 1B). Previous study has revealed that the tissues of PPTC7 knockout (KO) mice have markedly diminished mitochondrial content [24]. Considering the striking similarity in mitochondrial defects between PPTC7 KO and FBXL4 KO mice [24, 25], we conducted an investigation to determine whether PPTC7 regulates mitophagy via BNIP3/3L.

To validate our hypothesis, we first conducted co-immunoprecipitation (co-IP) assays and demonstrated that ectopically-overexpressed PPTC7 interacted with both BNIP3 and BNIP3L. In contrast, PPTC7 did not interact with other mitophagy cargo receptors including PHB2, NIPSNAP1, and FUNDC1, which underscores the highly specific interaction between PPTC7 and BNIP3/3L (Fig. 1C). Exogenous co-IP assay also demonstrated an interaction between PPTC7 and FBXL4 (Fig. 1D). Additionally, FLAG-FBXL4 immunoprecipitated endogenous BNIP3/3L and PPTC7 (Fig. 1E), while PPTC7-FLAG immunoprecipitated endogenous BNIP3/3L and FBXL4 (Fig. 1F). Finally, we demonstrated that PPTC7 interacted with BNIP3/3L at endogenous levels (Fig. 1G–I).

Given that BNIP3/3L and FBXL4 are all outer mitochondrial membrane proteins, we investigated whether PPTC7 is also located on the outer mitochondrial membrane. We further separated the nuclear, mitochondrial, and cytoplasmic fractions of HeLa cells using density-gradient centrifugation methods. PPTC7 is predominantly localized in the mitochondria (Fig. 1J), consistent with previously reported studies [20]. Then, we incubated the isolated mitochondrial fractions with proteinase K in the presence or absence of the detergent Triton X-100. Proteins associated with the outer mitochondrial membrane are expected to be protease-sensitive, whereas internal proteins are degraded only after disruption of the mitochondrial membrane by Triton X-100. We found that HSP60 (a mitochondrial matrix protein) and SMAC (a mitochondrial intermembrane space protein) were resistant to proteinase K, whereas TOMM70 (a mitochondrial outer membrane protein) was completely degraded. When proteinase K was added to the mitochondria fraction in the presence of Triton X-100, all the tested proteins were degraded (Fig. 1K). As to PPTC7, we noted that proteinase K moderately reduced PPTC7 protein levels, whereas PPTC7 was completely degraded when Triton X-100 was added, which indicates that a proportion of PPTC7 is located on the outer mitochondrial membrane. In addition, we validated that BNIP3/3L is localized to the outer mitochondrial membrane (Fig. 1K). We also detected the co-localization of PPTC7 with HSP60 and BNIP3/3L through immunofluorescence (IF) analysis (Supplementary Figs. 1A, 3C).

Collectively, these data indicate that PPTC7 specifically interacts with BNIP3/3L in cells.

### PPTC7 controls BNIP3/3L protein stability in a protein phosphatase activity-independent manner

**Fig. 2:**
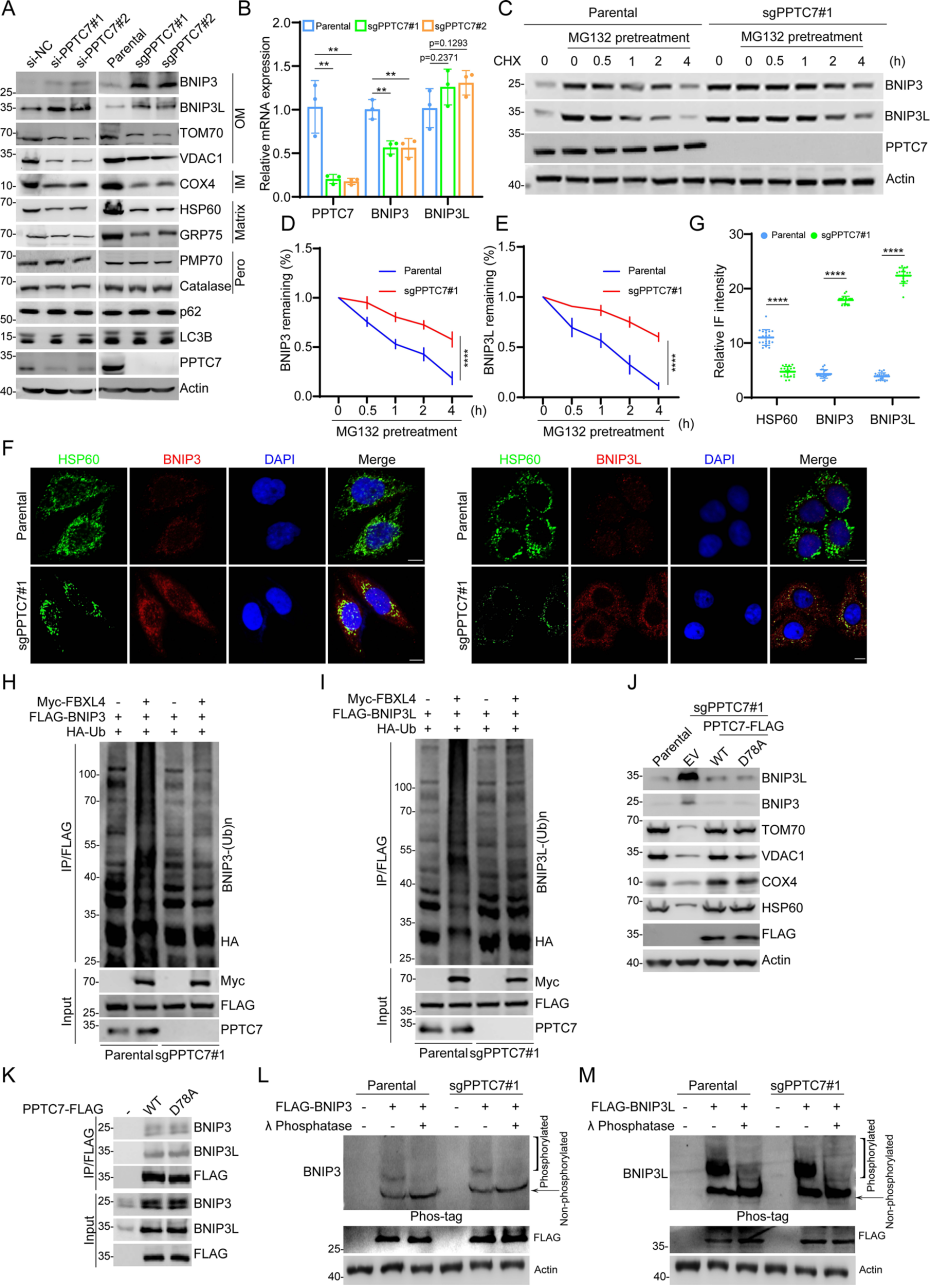
PPTC7 controls BNIP3/3L protein stability in a protein phosphatase activity-independent manner. **A** WB analysis of the indicated proteins in HeLa cells transfected with either negative control siRNA (siNC) or PPTC7-specific siRNAs, as well as from parental and PPTC7 knockout (KO) HeLa cells generated via LentiCRISPRv2. The indicated mitochondrial and cytoplasmic proteins were analyzed. Results are representative of three independent experiments. Additional replicates are shown in Supplementary Fig. 1C, and a quantitative analysis of these repetitions is presented in Supplementary Fig. 1D, E. **B** RT-qPCR measurement of PPTC7, BNIP3, and BNIP3L mRNA levels in parental and PPTC7 KO HeLa cells. Data are presented as means ± SD (n = 3). **C**–**E** WB analysis of BNIP3 and BNIP3L protein levels in parental and PPTC7 KO HeLa cells pretreated with DMSO or MG132 (20 μM, 5 h) before treatment with cycloheximide (CHX, 50 μg/mL). Samples were harvested at the indicated time points. Representative WBs are shown in (**C**), and protein stability curves are shown for BNIP3 (**D**) and BNIP3L (**E**) after normalization to Actin and the 0 h value. The other two repeats are shown in Supplementary Fig. 1H. Data represent means ± SD (n = 3). **F**, **G** Representative IF images of BNIP3, BNIP3L, and HSP60 in parental and PPTC7 KO HeLa cells. Nuclei were counterstained with DAPI. Scale bar, 10 μm. Quantitative analysis of IF signal intensity for HSP60, BNIP3, and BNIP3L is shown in (**G**). Data represent means ± SD (n = 20 cells per group). **H**, **I** WB analysis of in vivo ubiquitination assays in parental and PPTC7 KO HeLa cells transfected with plasmids expressing FLAG-BNIP3 (**H**), FLAG-BNIP3L (**I**), Myc-FBXL4, and HA-ubiquitin (HA-Ub). Immunoprecipitates were analyzed with anti-HA antibody to detect ubiquitinated proteins. **J** WB analysis of the indicated proteins in parental and PPTC7 KO HeLa cells stably overexpressing EV, PPTC7-WT-FLAG and PPTC7-D78A-FLAG mutant. **K** WB analysis of indicated proteins in the input and co-IP samples of anti-FLAG antibody obtained from 293 T cells transfected with PPTC7-WT-FLAG, or PPTC7-D78A-FLAG mutant. **L**, **M** Phos-tag SDS-PAGE (upper panel) or conventional SDS-PAGE (lower panel) analysis of BNIP3 and BNIP3L phosphorylation status in parental and PPTC7 KO HeLa cells transfected with FLAG-BNIP3 or FLAG-BNIP3L, followed by treatment with (+) or without (−) lambda phosphatase (λ phosphatase). WB was performed with anti-FLAG antibody to detect phosphorylated and non-phosphorylated forms. *P* values are calculated by the Two-way ANOVA test in (**B**, **D**, **E**, **G**). **P < 0.01; ****P < 0.0001.

Although PPTC7 interacts with FBXL4, PPTC7 protein levels were comparable between parental and FBXL4 KO HeLa cells, indicating that FBXL4 does not affect PPTC7 protein stability (Supplementary Fig. 1B). To examine whether PPTC7 regulates BNIP3/3L protein stability in a manner like FBXL4, we utilized siRNA-mediated knockdown (KD) or CRISPR/Cas9-mediated KO to deplete PPTC7 expression in HeLa cells. As shown in Fig. 2A, the depletion of PPTC7 resulted in a substantial elevation in the steady-state levels of endogenous BNIP3/BNIP3L. Additionally, there was a notable decline observed in the marker proteins within the submitochondrial compartments, such as TOM70 and VDAC1 in the outer membrane, COX4 in the inner membrane, and HSP60 and GRP75 in the matrix. Apart from mitochondria, BNIP3/BNIP3L are also found on peroxisomes where they play a crucial role in promoting pexophagy [26]. Pexophagy is a specific form of autophagy that selectively targets peroxisomes, and it is vital for maintaining the homeostasis of peroxisomes [27]. We observed that the depletion of PPTC7 did not reduce the protein levels of peroxisomal marker proteins (Catalase and PMP70), indicating that PPTC7 does not play a role in regulating pexophagy. We also examined the effect of PPTC7 on general autophagy and found that the loss of PPTC7 did not affect the protein levels of the autophagy markers LC3B and p62, indicating that PPTC7 does not affect the activation of general autophagy (Fig. 2A, Supplementary Fig. 1C–E). The impacts of PPTC7 on the protein levels of BNIP3/3L and other mitochondrial marker proteins were also observed in CCF-RC1 and Caki-1 cells (Supplementary Fig. 1F, G).

The mRNA levels of BNIP3 and BNIP3L were either decreased or remained unchanged in PPTC7-depleted cells compared to the control cells (Fig. 2B). This result indicated that the upregulation of BNIP3/3L proteins upon PPTC7 depletion is not achieved through the upregulation of their mRNA levels. It also suggests that PPTC7 may influence the protein levels of BNIP3/BNIP3L by modulating their half-life or through post-transcriptional regulation. Moreover, the half-life of BNIP3/BNIP3L was remarkably prolonged in PPTC7 KO cells (Fig. 2C–E, Supplementary Fig. 1H). Immunofluorescence (IF) analysis further revealed that the intensity of BNIP3/BNIP3L was markedly upregulated in PPTC7 KO cells, whereas the intensity of mitochondrial marker HSP60 was markedly decreased (Fig. 2F, G). Consistently, the endogenous ubiquitination levels of BNIP3/BNIP3L were markedly reduced in PPTC7 KO cells (Fig. 2H, I).

PPTC7 has been reported as a protein phosphatase localized in the mitochondrial matrix [20, 24]. PPTC7 was reported to dephosphorylate specific mitochondrial proteins, including the mitochondrial import complex protein TIMM50 [24]. To assess the significance of PPTC7’s phosphatase activity on BNIP3/BNIP3L and mitochondrial content, we introduced stable overexpression of either PPTC7-WT or its enzymatically inactive D78A mutant into PPTC7 KO cells. Unexpectedly, we observed that the reintroduction of PPTC7-WT or the D78A mutant into PPTC7 KO cells similarly reversed the previously observed accumulation of BNIP3/3L proteins and reduction in mitochondrial proteins caused by PPTC7 deficiency (Fig. 2J). We also demonstrated that PPTC7 and the D78A mutant immunoprecipitated BNIP3/3L to similar levels (Fig. 2K). To further assess the impact of PPTC7 ablation on the phosphorylation levels of BNIP3/3L, Phos-tag gels were used to detect changes in the electrophoretic mobility of phosphorylated proteins. We ectopically overexpressed FLAG-BNIP3 in the control or PPTC7 KO cells. We observed BNIP3 bands with higher levels of phosphorylation, and the mobility shifts of the phosphorylated BNIP3 were eliminated after treatment with λ phosphatase. However, the levels of phosphorylated FLAG-BNIP3 were comparable between the control and PPTC7 KO cells (Fig. 2L). Similar results were observed when we examined the phosphorylation levels of FLAG-BNIP3L in parental and PPTC7 KO cells (Fig. 2M).

Collectively, these data indicate that PPTC7 decreases the protein stability of BNIP3/3L in a manner independently of its protein phosphatase activity.

### PPTC7 deficiency activates basal mitophagy in a BNIP3/3L-dependent manner

**Fig. 3:**
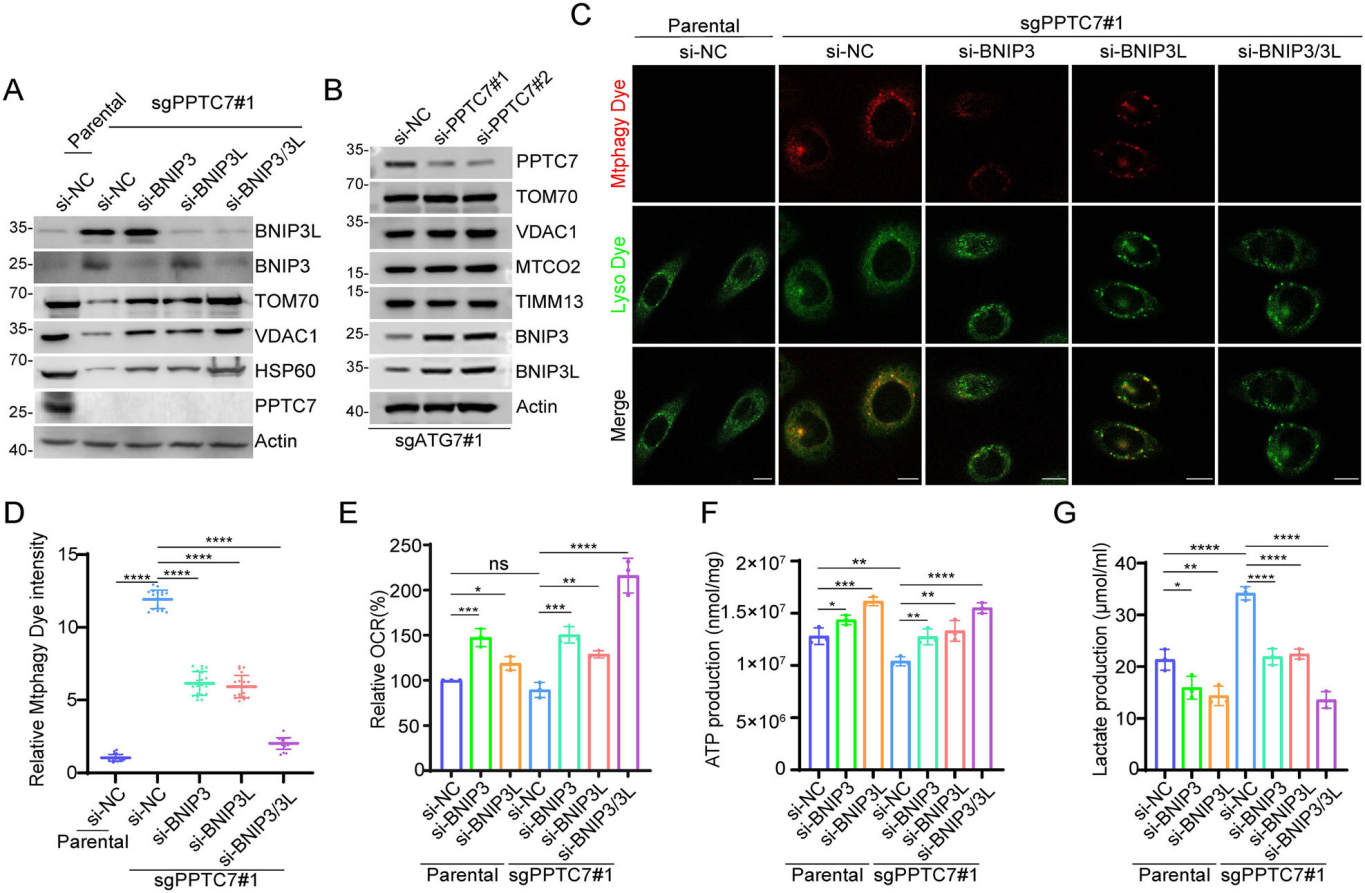
PPTC7 deficiency activates mitophagy, which is dependent on BNIP3/3L. (A) WB analysis of the indicated proteins in cell lysates from parental or PPTC7 KO HeLa cells transfected with the indicated siRNAs. (B) WB analysis of the indicated proteins in whole cell lysates (WCL) from ATG7-KO HeLa cells transfected with PPTC7-specific siRNAs or siNC. (C, D) Representative IF images of parental and PPTC7 KO HeLa cells transfected with either siNC or PPTC7-specific siRNAs. Cells were stained with Mtphagy Dye (to visualize mitophagy) and Lyso Dye (to label lysosomes). The relative fluorescence intensity of Mtphagy Dye in parental versus PPTC7 KO cells is quantified in (D). Scale bar, 10 μm. Data represent means ± SD (n = 20 cells per group). (E) Measurement of oxygen consumption rate (OCR) in parental and PPTC7 KO HeLa cells transfected with the indicated siRNAs or siNC, using an OCR Assay Kit. Data are shown as means ± SD (n = 3). (F) Quantification of intracellular ATP levels in parental and PPTC7 KO HeLa cells transfected with either siNC or PPTC7-specific siRNAs, using an ATP Production Assay Kit. Data are shown as means ± SD (n = 3). (G) Measurement of intracellular lactate levels in parental and PPTC7 KO HeLa cells transfected with siNC or PPTC7-specific siRNAs, using a Lactate Assay Kit. Data are shown as means ± SD (n = 3). *P* values are calculated by the Two-way ANOVA test in (D, E, F, G). *P < 0.05; **P < 0.01; ***P < 0.001; ****P < 0.0001; n.s. non-significant.

To examine whether the accumulation of BNIP3/3L proteins is responsible for the reduction of mitochondrial content, we performed individual or combined KD of BNIP3/3L in PPTC7 KO cells. Our results showed that KD of either BNIP3 or BNIP3L partly restored the downregulation of mitochondrial marker proteins caused by PPTC7 KO, whereas combined KD of BNIP3/3L completely restored it (Fig. 3A). ATG7 is essential for canonical autophagy. Consequently, we generated ATG7 KO cells and observed a marked accumulation of p62 and LC3B (Supplementary Fig. 2A, B). In ATG7 KO HeLa cells, we found an increase in the protein levels of BNIP3 and BNIP3L upon depletion of PPTC7 using siRNAs, while the levels of mitochondrial marker proteins remained unchanged (Fig. 3B). These results indicated that PPTC7 plays a crucial role in regulating mitochondrial content through the canonical autophagy pathway.

To monitor mitophagy, we utilized Mtphagy Dye, that accumulates in intact mitochondria and exhibits weak fluorescence under normal conditions. Upon induction of mitophagy, damaged mitochondria fuse with lysosomes, leading to strong fluorescence emission from Mtphagy Dye [28]. We found that PPTC7 KO significantly increased mitophagy signals. KD of BNIP3 or BNIP3L alone partially blocked the activation of mitophagy, whereas combined KD of BNIP3/3L completely blocked it (Fig. 3C, D). Additionally, we evaluated several mitochondrial metabolic indexes to assess mitochondrial functions. PPTC7 KO resulted in decreased rates of oxygen consumption (OCR) and ATP production, along with an increase in lactate production. Notably, combined KD of BNIP3/BNIP3L effectively reversed the observed effects in PPTC7 KO HeLa cells (Fig. 3E–G).

Collectively, these data indicate that PPTC7 deficiency induces hyperactive mitophagy due to BNIP3/3L accumulation.

### PPTC7 deficiency activates BNIP3/3L-dependent mitophagy in the *Pptc7* KO mouse model

**Fig. 4:**
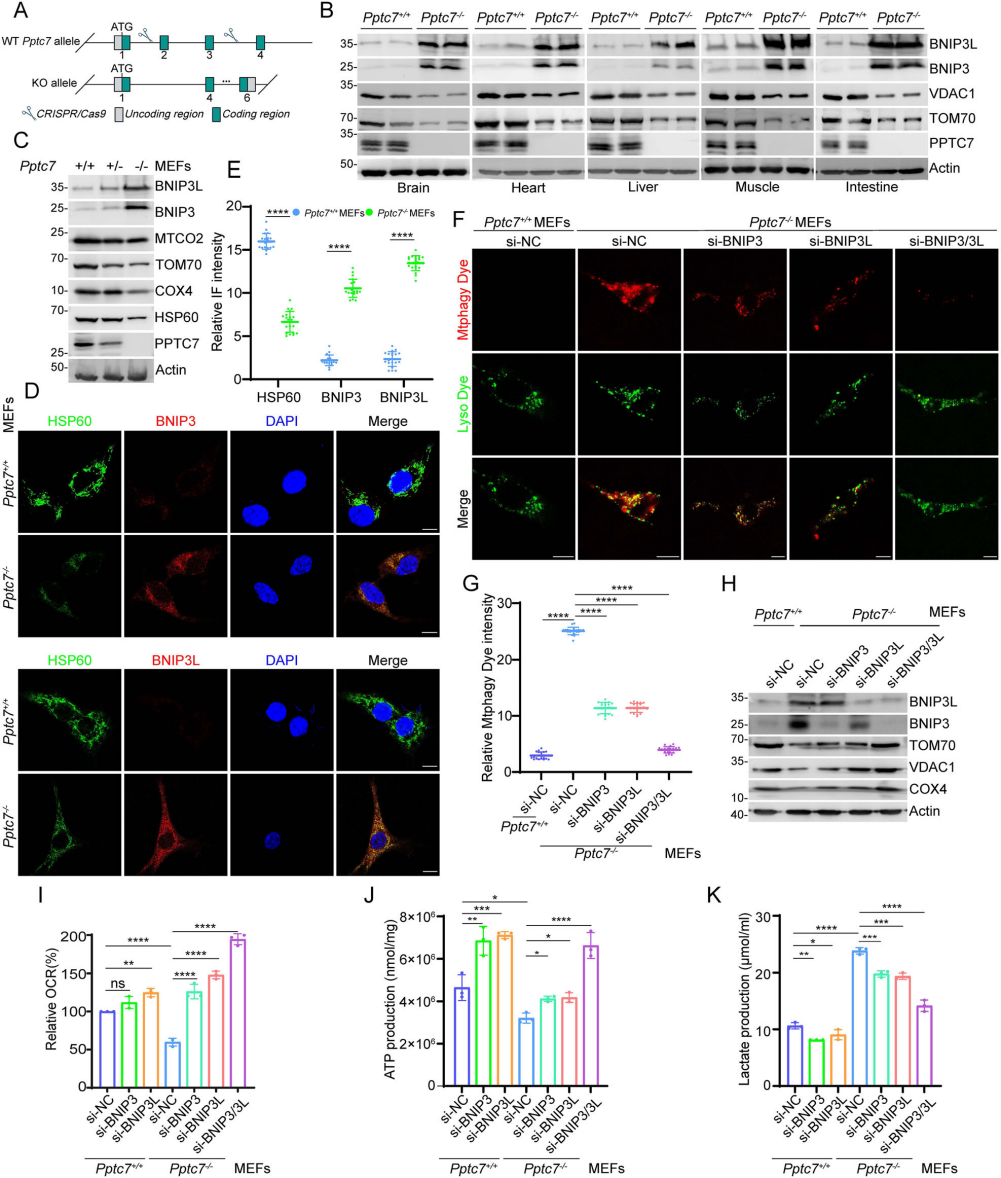
BNIP3/3L-dependent mitophagy was abnormally activated in *Pptc7* KO mouse model. **A** Schematic representation of the strategy used to generate Pptc7 knockout (KO) mice using CRISPR/Cas9 technology. **B** WB analysis of the indicated protein in the input from various tissues, including brain, heart, liver, muscle, and intestine, of *Pptc7*^*+/+*^ and *Pptc7*^*−/−*^ mice at embryonic day 20 (E20). Actin was used as a loading control. **C** WB analysis of the indicated proteins in WCL from *Pptc7*^*+/+*^, *Pptc7*^*+/−*^ and *Pptc7*^*−/−*^ MEFs. **D**, **E** Representative IF images of *Pptc7*^*+/+*^ and *Pptc7*^*−/−*^ MEFs stained with BNIP3, BNIP3L, HSP60 (mitochondrial marker), and DAPI (nuclear marker). The relative fluorescence intensity of HSP60, BNIP3, and BNIP3L was quantified and shown in (**E**). Data were shown as means ± SD (n = 20 cells per group). Scale bar, 10 μm. **F**, **G** Representative IF images of *Pptc7*^*+/+*^ and *Pptc7*^*−/−*^ MEFs transfected with negative control siRNA (siNC) or specific siRNAs targeting BNIP3 and BNIP3L. Cells were stained with Mtphagy Dye and Lyso Dye. Quantification of Mtphagy Dye intensity is shown in (**G**). Scale bar, 10 μm. Data were shown as means ± SD (n = 20 cells per group). **H** WB analysis of mitochondrial and autophagy-related proteins in WCL from *Pptc7*^*+/+*^ and *Pptc7*^*−/−*^ MEFs transfected with siNC or siRNAs targeting BNIP3 and BNIP3L. **I** Measurement of OCR in *Pptc7*^*+/+*^ and *Pptc7*^*−/−*^ MEFs transfected with siNC or the indicated siRNAs using an OCR assay kit. Data are shown as means ± SD (n = 3). **J** Quantification of intracellular ATP production in *Pptc7*^*+/+*^ and *Pptc7*^*−/−*^ MEFs transfected with siNC or the indicated siRNAs, measured using an ATP production assay kit. **K** Measurement of intracellular lactate levels in *Pptc7*^+/+^ and *Pptc7*^−/−^ MEFs transfected with siNC or the indicate siRNAs, using a Lactate Assay Kit. Data are shown as means ± SD (n = 3). *P* values are calculated by the Two-way ANOVA test in (**E**, **G**, **I**, **J**, **K**). *P < 0.05; **P < 0.01; ***P < 0.001; ****P < 0.0001; n.s. non-significant.

To gain a better understanding of the pathophysiological roles of PPTC7 on BNIP3/3L proteins and mitophagy in vivo, we generated *Pptc7* knockout mouse model (Fig. 4A). Consistent with a previous study showing that *Pptc7* KO in mice caused fully penetrant lethality, no mice survived at P10 in our experimental settings [24]. Thus, we collected multiple tissues, including brain, heart, liver, muscle, and intestine from embryonic day 20 (E20) mice. WB analysis indicated that *Pptc7*^−*/*−^ mice tissues exhibited varying degrees of upregulation in BNIP3/3L protein levels, compared to those of wild-type (WT) mice. Additionally, mitochondrial proteins were inevitably downregulated across all the examined tissues (Fig. 4B).

We next prepared mouse embryonic fibroblasts (MEFs). In homozygous *Pptc7*^*−/−*^ MEFs, BNIP3/3L protein levels were markedly elevated, while mitochondrial marker protein levels were reduced compared to WT or heterozygous *Pptc7*^*+/−*^ MEFs (Fig. 4C–E). *Pptc7*^−/−^ MEFs exhibited a high level of basal mitophagy, which was almost undetectable in WT MEFs. Notably, KD of BNIP3 or BNIP3L alone partially inhibited Pptc7 KO-induced mitophagy activation, while combined KD of BNIP3/3L completely blocked it (Fig. 4F–H). To further confirm the critical role of PPTC7 in mitophagy, *Pptc7*^*+/+*^ and *Pptc7*^*−/−*^ MEFs were examined by Transmission Electron Microscopy (TEM). The number of mitophagic vacuoles was substantially higher in *Pptc7*^*−/−*^ MEFs compared to *Pptc7*^*+/+*^ MEFs. Additionally, the mitochondrial area in *Pptc7*^−/−^ MEFs was significantly reduced compared to that in *Pptc7*^*+/+*^ MEFs (Supplementary Fig. 3A, B). Furthermore, *Pptc7*^*−/−*^ MEFs displayed decreased rates of OCR and ATP production, along with an increase in lactate production. Importantly, these effects were largely reversed by combined KD of BNIP3/3L (Fig. 4I–K).

Collectively, these data indicate that PPTC7 deficiency in mice induces hyperactive mitophagy due to BNIP3/3L accumulation.

### PPTC7 is an essential co-factor of the SCF^FBXL4^ E3 ubiquitin ligase complex

**Fig. 5:**
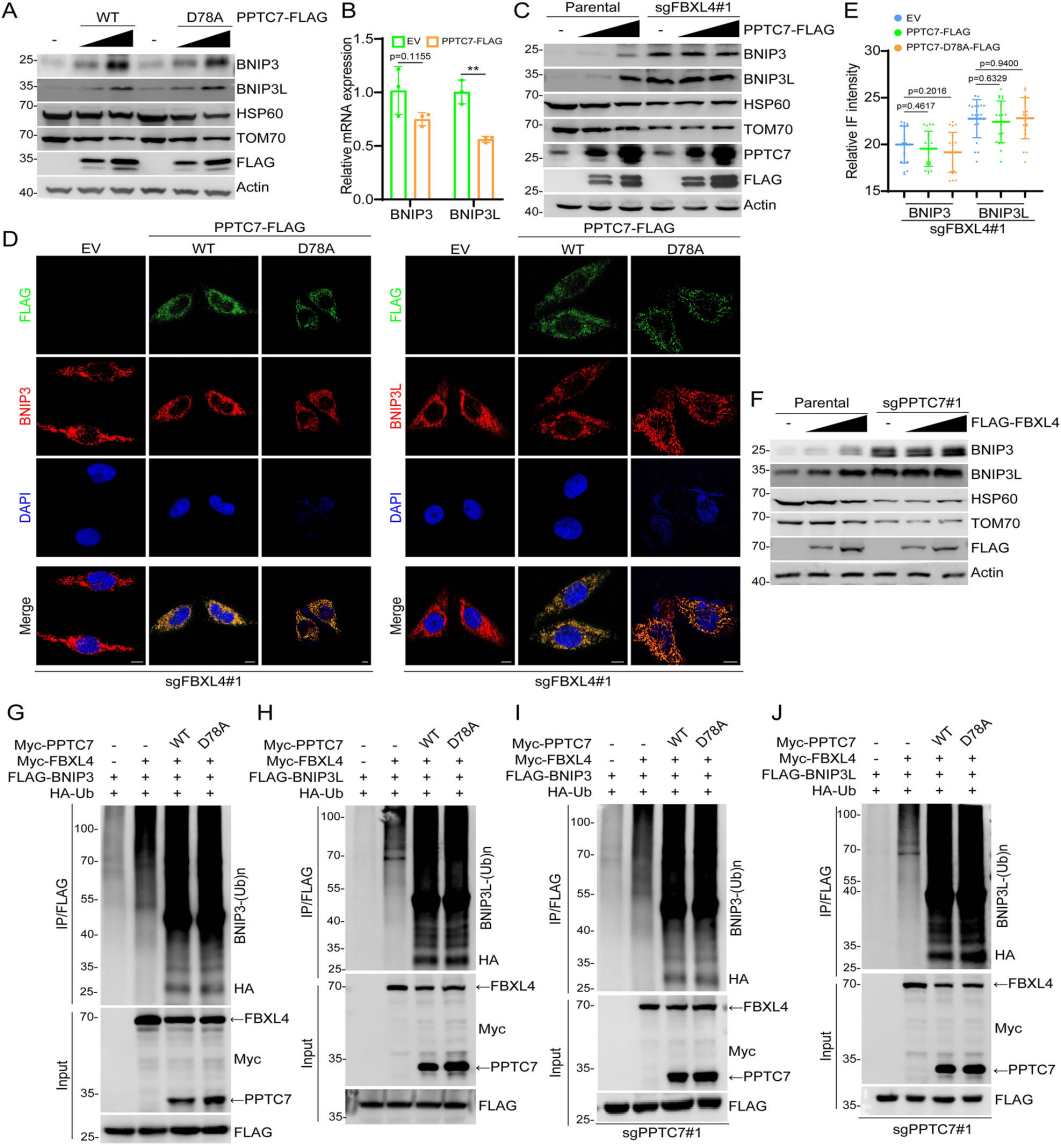
PPTC7 is an essential co-factor of SCF^FBXL4^ E3 ubiquitin ligase complex. **A** WB analysis of the indicated proteins in the input from parental HeLa cells stably overexpressing EV, PPTC7-WT-FLAG, or PPTC7-D78A-FLAG mutant. **B** RT-qPCR measurement of BNIP3 and BNIP3L mRNA levels in HeLa cells stably overexpressing EV or PPTC7-FLAG. Data are shown as means ± SD (n = 3). **C** WB analysis of the indicated proteins in WCL from FBXL4 KO HeLa cells stably overexpressing EV or PPTC7-FLAG. **D**, **E** Representative IF images from FBXL4 KO HeLa cells stably overexpressing EV, PPTC7-WT-FLAG, or PPTC7-D78A-FLAG mutant, stained with BNIP3 (or BNIP3L), HSP60, and DAPI. Scale bar, 10 μm. The relative intensity of HSP60, BNIP3, and BNIP3L was quantified and shown in (**E**). Data were shown as means ± SD (n = 20 cells per group). **F** WB analysis of the indicated proteins in WCL from PPTC7 KO HeLa cells stably overexpressing EV or FLAG-FBXL4. **G**, **H** WB of the products of in vivo ubiquitination assays from parental HeLa cells transfected with the indicated plasmids. **I**, **J** WB of the products of in vivo ubiquitination assays from PPTC7 KO HeLa cells transfected with the indicated plasmids. *P* values are calculated by the Two-way ANOVA test in (**B**, **E**). **P < 0.01.

As PPTC7 does not contain any domains related to ubiquitin-proteasome system, it does not seem likely to directly promote the ubiquitination and degradation of BNIP3/3L. The aforementioned results indicated that depletion of PPTC7 resulted in BNIP3/3L accumulation, resembling the phenotypes observed in FBXL4-KO cells. Since PPTC7 interacts with FBXL4, we investigated whether PPTC7 acts as a co-factor of the SCF^FBXL4^ E3 ubiquitin ligase complex to regulate BNIP3/3L protein stability. Surprisingly, similar to the outcomes from the PPTC7 depletion experiments, we observed that the exogenous overexpression of PPTC7-WT or the catalytically inactive D78A mutant in parental HeLa cells both markedly increased the steady-state levels of endogenous BNIP3/3L and simultaneously reduced mitochondrial content (Fig. 5A, Supplementary Fig. 3C, D). The mRNA levels of BNIP3/3L were either reduced or remained unchanged in PPTC7-overexpressed cells (Fig. 5B). These results underscored the critical role of maintaining precise control over PPTC7 protein levels in regulating the abundance of BNIP3/3L. In contrast, ectopic overexpression of PPTC7-WT in FBXL4 KO cells showed no impact on the protein levels of BNIP3/3L and mitochondrial content (Fig. 5C). IF analysis also revealed that the overexpression of PPTC7-WT or the D78A mutant in FBXL4-KO cells failed to reverse the BNIP3/3L accumulation caused by FBXL4 deficiency (Fig. 5D, E).

Similarly, ectopic overexpression of FBXL4 in parental HeLa cells markedly increased the steady-state levels of endogenous BNIP3/3L and simultaneously reduced mitochondrial content (Fig. 5F, Supplementary Fig. 3E, G). However, ectopic overexpression of FBXL4 in PPTC7 KO cells did not affect the protein levels of BNIP3/3L and mitochondrial content (Fig. 5F, Supplementary Fig. 3G, H). Finally, we showed that co-expression of either PPTC7-WT or the D78A mutant further enhanced the FBXL4-induced ubiquitination of BNIP3/3L in parental and PPTC7 KO cells (Fig. 5G–J).

Collectively, these data suggest that PPTC7 acts as an essential co-factor of the SCF^FBXL4^ E3 ubiquitin ligase complex to facilitate the ubiquitination and degradation of BNIP3/3L.

## Discussion

**Fig. 6:**
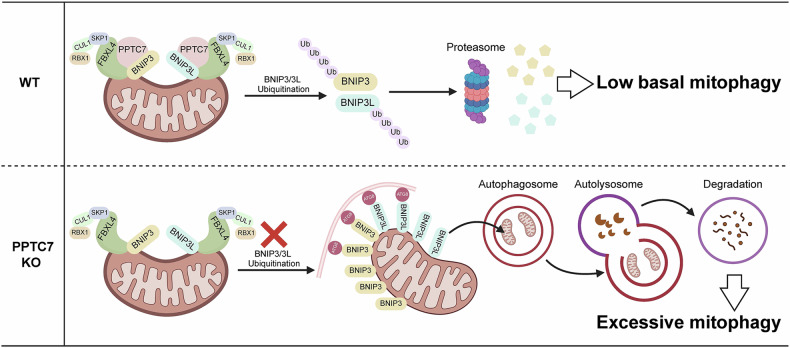
A diagram illustrating the role of PPTC7 as an essential co-factor in the SCFF^BXL4^ ubiquitin ligase complex, which restricts BNIP3/3L-dependent mitophagy.

Using in vitro cell culture and *Pptc7* knockout mouse model, we demonstrate that PPTC7 acts as an essential co-factor of the SCF^FBXL4^ complex to facilitate ubiquitin-proteasomal degradation of BNIP3/3L, thereby keeping basal mitophagy at a very low level (Fig. 6). This active regulation may allow a rapid mitophagy response under certain conditions, by disrupting SCF^FBXL4/PPTC7^-mediated BNIP3/3L degradation. However, the upstream molecular events and physiological functions underlying these are still elusive.

Our results are also corroborated by a recent study showing that *Pptc7* KO mice caused hyperactive mitophagy in a BNIP3/3L-dependent manner [29]. During the preparation of this manuscript, a published study also reported that PPTC7 acts as a mitophagy sensor to control BNIP3/3L degradation to regulate mitophagy [29]. This study furthermore demonstrated that Pptc7 KO-induces perinatal lethality can be rescued by Bnip3L KO, indicating Bnip3L-mediated mitophagy may be a critical downstream event in vivo [30]. One unexpected phenomenon we observed is that overexpression of PPTC7 also resulted in the stabilization of BNIP3/3L and induction of mitophagy, similar to the phenotypes seen in PPTC7 knockout. It is tempting to speculate that the subunit stoichiometry is a critical determinant of the correct function of the SCF^FBXL4/PPTC7^ complex, although the molecular details warrant further investigation. It also suggests that simply upregulating the protein levels of PPTC7 is insufficient to suppress BNIP3/3L-dependent mitophagy; both PPTC7 and FBXL4 might need to be co-regulated.

In this study, we observed that a significantly lower mRNA of BNIP3/3L after KO of PPTC7. Similar results were observed in FBXL4 KO cells [20]. The underlying mechanisms for the significantly lower mRNA levels of BNIP3/3L following the KO of PPTC7 or FBXL4 are not fully understood at present. However, we propose that this could represent a form of negative feedback regulation at the level of gene expression. Specifically, when the protein levels of BNIP3/3L rise sharply, it may activate mechanisms that suppress their own mRNA synthesis or stability, serving as a buffering system to prevent further protein accumulation. Such negative feedback loops may be critical for maintaining cellular homeostasis and preventing excessive protein production, although the specific mechanisms remain to be explored.

PPTC7 is reported as a mitochondrial matrix phosphatase in yeast and mammalian cells. Using submitochondrial fraction method, we demonstrated that a proportion of PPTC7 is located on the outer mitochondrial membrane, it explains why PPTC7 can interact with outer mitochondrial membrane protein FBXL4 and BNIP3/3L. We also showed that reintroduction of PPTC7-WT or its enzymatically inactive mutant into PPTC7 KO cells comparably reversed the accumulation of BNIP3/3L proteins. Moreover, the phosphorylation levels of BNIP3/3L were comparable between parental and PPTC7 KO cells as judged by phos-tag gels. These results indicate that the phosphatase activity may be dispensable for its role in regulating BNIP3/3L turnover. PPTC7 has a homologous protein in yeast, but FBXL4 does not have a homologous protein in yeast. An unpublished study in a master’s thesis showed that RNAi knockdown of CG12091 (the homolog PPTC7 protein of Drosophila) resulted in drastic changes in mitochondrial morphology and neurodegeneration in Drosophila. Furthermore, overexpression of CG12091-WT or the enzymatically inactive mutant CG12091mu significantly impeded the development of the Drosophila compound eye and abnormal mitochondria were observed in CG12091-WT or CG12091mu overexpressed photoreceptor cells [31]. From an evolutionary perspective, we speculate that SCF^FBXL4/PPTC7^-mediated mitophagy regulation may emerge after yeast but before Drosophila.

## Materials and Methods

### Cell culture

293 T, HeLa, and MEF cells were cultured in Dulbecco’s Modified Eagle’s Medium (DMEM) supplemented with 10% fetal bovine serum (FBS). Caki-1 and CCF-RC1 cells were cultured in Roswell Park Memorial Institute 1640 Medium (RPMI-1640) supplemented with 10% FBS. All cells were cultured at 37 °C under 5% CO_2_ in a humidified incubator. DNA fingerprinting and PCR were performed to verify the authenticity of the cell lines and to ensure they were free of mycoplasma infection. Transient transfection was conducted using EZ Trans (Shanghai Life-iLab Biotech) or Lipofectamine 8000 (Beyotime). For lentiviral transfection, pLVX overexpression plasmids and virus-packing constructs were transfected into 293 T cells. The viral supernatant was collected after 48 h. The cells were then infected with the viral supernatant in the presence of polybrene (8 µg/ml) and subsequently selected in growth media containing puromycin (1.5 μg/ml). The sequences of gene-specific siRNAs are provided in Supplementary Table 1.

### Antibodies and chemicals

The information of antibodies and chemicals used in this study is listed in Supplementary Table 2, 3.

### Gene KO cell line generation

To knockout *PPTC7*, *FBXL4*, or *ATG7* genes in human cells, CRISPR/Cas9 protocols were employed. The sgRNAs were designed using an online CRISPR design tool (http://crispr.mit.edu) and were then subcloned into the LentiCRISPRv2 vector from Dr. Feng Zhang’s lab. Guide RNA containing the target sequence and virus-packing constructs were transfected into 293 T cells. The viral supernatant was collected after 48 h. HeLa, Caki-1, and CCF-RC1 cells were then infected with the viral supernatant in the presence of polybrene (8 µg/ml). The cells were selected with puromycin (2 µg/ml) for a period of 3 to 7 days. Successful KO of each gene was verified via Western blot (WB) analysis. The sequences of gene-specific sgRNAs are listed in Supplementary Table 1.

### In vivo ubiquitination assays

For ubiquitination analysis, HA-tagged ubiquitin and other indicated plasmids were co-transfected into 293 T or HeLa cells. 36 h After transfection, MG132 was added to the medium for 4–6 h before cell harvesting. Cells were collected, lysed, and boiled in 1% SDS buffer (Tris-HCl, pH 7.5, 0.5 mM EDTA, 1 mM DTT) for 10 min. Immunoprecipitation was performed in 10-fold diluted lysates with 0.5% NP-40 buffer, and the ubiquitination levels of BNIP3 or BNIP3L were detected via WB.

### RNA isolation and quantitative real-time reverse transcription PCR (qRT-qPCR)

Total RNA was isolated from cells using the TransZol Up reagent (TRANS) following the manufacturer’s instructions. Concentrations and purity of RNAs were determined by measuring the absorption of ultra-violet lights using a NanoDrop spectrophotometer (Thermo). cDNAs were reversed-transcribed using a HiScript III RT SuperMix for qPCR (Vazyme), followed by amplification of cDNA using ChamQ SYBR qPCR Master Mix (Vazyme). The relative mRNA levels of genes were quantified using the 2-ΔΔCt method, with normalization to Actin. The sequences of primers are listed in Supplementary Table 1.

### IF and confocal microscopy

HeLa or MEF cells were seeded on glass coverslips in 12-well plates and harvested at 80% confluence. The cells were washed with PBS and fixed with 4% paraformaldehyde in PBS at room temperature (RT) for 30 min. After permeabilization with 0.1% Triton X-100 for 30 min and then in the blocking solution (PBS plus 5% donkey serum), for 1 h at RT. The cells were then incubated with primary antibodies at 4 °C overnight. After washing with PBST buffer, fluorescence-labeled secondary antibodies were applied. DAPI was used to stain nuclei. The glass coverslips were mounted on slides and imaged using a confocal microscope (LSM880, Zeiss) with a 63*/1.4NA Oil PSF Objective. Quantitative analyses were performed using ImageJ software.

### Isolation of nucleus, cytoplasm, and mitochondria

HeLa cells were prepared for nuclear, cytoplasmic, and mitochondrial extraction using density-gradient centrifugation. Briefly, 5 × 10^6^ HeLa cells were washed three times with PBS and then suspended by using hypotonic solution (140 mM KCl, 10 mM EDTA, 5 mM MgCl_2_, 20 mM HEPES (pH 7.4), and the protease inhibitor). Next, the cells were ground with a glass homogenizer in an ice bath for 25 strokes. Nuclear, cytoplasmic, and mitochondrial fractions were separated through differential centrifugation (800 × g, 10 min, 4 °C and 12,000 × g, 35 min, 4 °C). The supernatant (cytoplasmic fraction) and pellet (mitochondrial fraction) were collected, and the pellet was further washed with wash buffer (800 mM KCl, 10 mM EDTA, 5 mM MgCl_2_, and 20 mM HEPES (pH 7.4), and the protease inhibitor) for three times to yield the final mitochondrial fraction. To confirm that pure extracts were obtained, the mitochondrial, nuclear, and cytoplasmic fractions were separated by SDS-PAGE, and the presence of mitochondrial COX4, nuclear Histone H3, and cytoplasmic GAPDH was detected via WB.

### Mitochondrial protein localization assays

Mitochondria were purified using the methods described above. The control group was left untreated. The second group was treated with proteinase K (3 μM). The third group was treated with proteinase K (3 μM) and 0.5% Triton-X-100 solution. Three groups of samples were placed in a 37 °C water bath for 30 min. The samples were prepared and subsequently analyzed by WB.

### Oxygen consumption assays

Oxygen consumption rate (OCR) was measured under basal conditions in the presence of the mitochondrial inhibitor oligomycin (0.25 μM, Calbiochem) at 37 °C. OCR was calculated based on the changes induced by oligomycin in comparison to the basal rates. HeLa and MEF cells were seeded at a density of 2 × 10^4^ cells in the cell culture microplate. The total protein of each well was determined by Bradford assay and used as the reference to normalize the OCR.

### ATP measurement assays

HeLa or MEF cells were seeded in 6-well plates at 2 × 10^6^ cells per well and cultured overnight.

Then, the cells were transfected with the indicated siRNAs. 48 h after transfection, the cells were collected for determination of ATP production using the ATP assay kit (Beyotime). The ATP assay kit is developed based on the principle that firefly luciferase catalyzes the production of luminescence from luciferin, which requires energy provided by ATP. When both firefly luciferase and luciferin are in excess, within a certain concentration range, the luminescence produced is proportional to the concentration of ATP. This allows for highly sensitive detection of ATP concentrations in solutions. According to the manual of the ATP assay kit (Beyotime), RLU were measured using a microplate absorbance reader (Bio-Rad).

### Lactate measurement assays

HeLa or MEF Cells were seeded into 6-well plates at a density of 1 × 10^5^ cells per well and cultured overnight. According to the manual of the lactate acid assay kit (Solarbio), lactate was converted to pyruvate by lactate dehydrogenase, which simultaneously reduced NAD^+^ to form NADH and H^ +^. The H^+^ was then transferred to PMS (phenazine methosulfate) to generate reduced PMSH2, which in turn reduced MTT to form a purple compound with a characteristic absorption peak at 570 nm. The resulting color was measured at 570 nm using a microplate absorbance reader (Bio-Rad).

### Generation and breeding of *Pptc7* Cas9-KO mice

*Pptc7* CRISPR/Cas9-KO mice were designed and generated by GemPharmatech Co., Ltd. In brief, Cas9 mRNA was in vitro transcribed with mMESSAGE T7 Ultra Kit (Ambion) according to the manufacturer’s instructions, and subsequently purified using the MEGAclearTM kit (Thermo) Cas9 sgRNA was in vitro transcribed using the MEGAshortscript kit (Thermo) and subsequently purified using MEGAclearTM kit. The transcribed Cas9 mRNA and sgRNA as well as a 200 base pairs single-stranded oligo deoxy nucleotide (ssODN) were co-injected into zygotes of C57BL/6 J mice. Obtained F0 mice were validated by PCR and Sanger sequencing. The F0 mice with expected point mutation were chosen and crossed with C57BL/6 J mice to produce F1 mice. Genotyping was performed by PCR analysis of tail DNA. The sequences of sgRNAs are listed in Supplementary Table 1. Mice were maintained under a 12 h/12 h light/dark cycle at 22–25 °C and 40–50% humidity with standard food and water available ad libitum.

### MEFs generation and immortalization

Timed pregnant female mice at embryonic day 12.5 to 14.5 were sacrificed, and the embryos were carefully dissected to remove the cerebrum, internal organs, and limbs. The remaining tissues were cut into small pieces and treated with trypsin-EDTA (0.25%) for 10 min at 37 °C. The trypsin was neutralized with DMEM, a complete medium supplemented with 10% FBS and 1% penicillin/streptomycin. The culture media were changed every 2–3 days until the cells reached confluence. To immortalize MEFs, they were passaged up to approximately 10 times before infection with lentiviral vectors expressing the SV40 large T-antigen. Stable transduction was achieved with puromycin selection. The successful integration of the immortalizing gene was confirmed through Sanger sequencing and WB analysis.

### Embryonic mouse tissue isolation

Around embryonic day 20, the pregnant female mouse was sacrificed, and the abdominal cavity was carefully opened under sterile conditions using sterile scissors and forceps. Using sterile forceps, each embryo was carefully extracted from the uterus. Specific tissues of interest were removed.

### Transmission electron microscopy

Discard the culture medium from the MEFs and add 2.5% room temperature glutaraldehyde fixative. Fix at room temperature for about 5 min, then use a cell scraper to gently scrape the cells off in one direction. Use a Pasteur pipette to transfer the cell suspension into a centrifuge tube and centrifuge at 2000 rpm for 2 min. After centrifugation, discard the fixative, add fresh electron microscopy fixative, and gently lift the cell pellet, suspending it in the new fixative. Fix at room temperature in the dark for 30 min. After dehydration with gradient ethanol and acetone, the samples were sequentially embedded with a 1:1 ratio of acetone: EMBed 812 for 2–4 h at 37 °C, a 1:2 ratio of acetone: EMBed 812 overnight at 37 °C and pure EMBed 812 for 5–8 h at 37 °C. The samples were moved into a 65 °C oven for polymerization for more than 48 h. Then, the sample blocks were sliced into 60-nm sections with an ultramicrotome for staining. After drying overnight at room temperature, the samples were imaged using a transmission electron microscope (CM120, Philips).

### Statistical analysis

Band intensities of WB results were calculated by ImageJ using the manufacturer’s instructions. Statistical analysis was performed using Prism 8.0 (GraphPad Software, Inc., San Diego, CA, USA) and Excel (Microsoft Corp., Redmond, CA, USA). Pooled results were expressed as the mean ± SEM. Comparisons between groups were made via One-way analysis of variance (ANOVA) or Two-way ANOVA. Statistical significance was set at *P* ≤ 0.05; ns no significance; *P < 0.05; **P < 0.01; ***P < 0.001; ****P < 0.0001.

## Supplementary information

Supplementary Figures

Supplementary Tables

Original Data

## Data Availability

All data needed to evaluate the conclusions in the paper are present in the paper and/or the Supplementary Materials.

## References

[1] Palikaras K, Lionaki E, Tavernarakis N. Mechanisms of mitophagy in cellular homeostasis, physiology and pathology. Nat Cell Biol. 2018;20:1013–22. 30154567 10.1038/s41556-018-0176-2

[2] Onishi M, Yamano K, Sato M, Matsuda N, Okamoto K. Molecular mechanisms and physiological functions of mitophagy. EMBO J. 2021;40:e104705. 33438778 10.15252/embj.2020104705 PMC7849173

[3] Pickles S, Vigie P, Youle RJ. Mitophagy and Quality Control Mechanisms in Mitochondrial Maintenance. Curr Biol. 2018;28:R170–R85. 29462587 10.1016/j.cub.2018.01.004 PMC7255410

[4] Picca A, Faitg J, Auwerx J, Ferrucci L, D’Amico D. Mitophagy in human health, ageing and disease. Nat Metab. 2023;5:2047–61. 38036770 10.1038/s42255-023-00930-8 PMC12159423

[5] Doxaki C, Palikaras K. Neuronal Mitophagy: Friend or Foe? Front Cell Dev Biol. 2020;8:611938. 33537304 10.3389/fcell.2020.611938 PMC7848077

[6] Wilhelm LP, Ganley IG. Eating your mitochondria-when too much of a good thing turns bad. EMBO J. 2023;42:e114542. 37272260 10.15252/embj.2023114542 PMC10308347

[7] Newman LE, Shadel GS. Pink1/Parkin link inflammation, mitochondrial stress, and neurodegeneration. J Cell Biol. 2018;217:3327–9. 30154188 10.1083/jcb.201808118 PMC6168260

[8] Harper JW, Ordureau A, Heo JM. Building and decoding ubiquitin chains for mitophagy. Nat Rev Mol Cell Biol. 2018;19:93–108. 29358684 10.1038/nrm.2017.129

[9] McWilliams TG, Prescott AR, Montava-Garriga L, Ball G, Singh F, Barini E, et al. Basal Mitophagy Occurs Independently of PINK1 in Mouse Tissues of High Metabolic Demand. Cell Metab. 2018;27:439–49.e5. 29337137 10.1016/j.cmet.2017.12.008 PMC5807059

[10] Killackey SA, Philpott DJ, Girardin SE. Mitophagy pathways in health and disease. J Cell Biol. 2020;219:e202004029. 10.1083/jcb.202004029 PMC7594502 32926082

[11] Ganley IG, Simonsen A Diversity of mitophagy pathways at a glance. J Cell Sci. 2022;135:jcs259748. 10.1242/jcs.259748 PMC10656428 36504076

[12] Field JT, Gordon JW. BNIP3 and Nix: Atypical regulators of cell fate. Biochim Biophys Acta Mol Cell Res. 2022;1869:119325. 35863652 10.1016/j.bbamcr.2022.119325

[13] Cao Y, Zheng J, Wan H, Sun Y, Fu S, Liu S, et al. A mitochondrial SCF-FBXL4 ubiquitin E3 ligase complex degrades BNIP3 and NIX to restrain mitophagy and prevent mitochondrial disease. EMBO J. 2023;42:e113033. 36896912 10.15252/embj.2022113033 PMC10308365

[14] Elcocks H, Brazel AJ, McCarron KR, Kaulich M, Husnjak K, Mortiboys H, et al. FBXL4 ubiquitin ligase deficiency promotes mitophagy by elevating NIX levels. EMBO J. 2023;42:e112799. 37102372 10.15252/embj.2022112799 PMC10308357

[15] Nguyen-Dien GT, Kozul KL, Cui Y, Townsend B, Kulkarni PG, Ooi SS, et al. FBXL4 suppresses mitophagy by restricting the accumulation of NIX and BNIP3 mitophagy receptors. EMBO J. 2023;42:e112767. 37161784 10.15252/embj.2022112767 PMC10308361

[16] Chen Y, Jiao D, Liu Y, Xu X, Wang Y, Luo X, et al. FBXL4 mutations cause excessive mitophagy via BNIP3/BNIP3L accumulation leading to mitochondrial DNA depletion syndrome. Cell Death Differ. 2023;30:2351–63. 37568009 10.1038/s41418-023-01205-1 PMC10589232

[17] Gao K, Xu X, Wang C. FBXL4 mutation-caused mitochondrial DNA depletion syndrome is driven by BNIP3/BNIP3L-dependent excessive mitophagy. Trends Mol Med. 2024;30:113–6. 38123379 10.1016/j.molmed.2023.11.017

[18] Bonnen PE, Yarham JW, Besse A, Wu P, Faqeih EA, Al-Asmari AM, et al. Mutations in FBXL4 cause mitochondrial encephalopathy and a disorder of mitochondrial DNA maintenance. Am J Hum Genet. 2013;93:471–81. 23993193 10.1016/j.ajhg.2013.07.017 PMC3769921

[19] Gai X, Ghezzi D, Johnson MA, Biagosch CA, Shamseldin HE, Haack TB, et al. Mutations in FBXL4, encoding a mitochondrial protein, cause early-onset mitochondrial encephalomyopathy. Am J Hum Genet. 2013;93:482–95. 23993194 10.1016/j.ajhg.2013.07.016 PMC3769923

[20] Guo X, Niemi NM, Hutchins PD, Condon SGF, Jochem A, Ulbrich A, et al. Ptc7p Dephosphorylates Select Mitochondrial Proteins to Enhance Metabolic Function. Cell Rep. 2017;18:307–13. 28076776 10.1016/j.celrep.2016.12.049 PMC5234840

[21] Tsherniak A, Vazquez F, Montgomery PG, Weir BA, Kryukov G, Cowley GS, et al. Defining a Cancer Dependency Map. Cell. 2017;170:564–76.e16. 28753430 10.1016/j.cell.2017.06.010 PMC5667678

[22] Bayraktar EC, La K, Karpman K, Unlu G, Ozerdem C, Ritter DJ, et al. Metabolic coessentiality mapping identifies C12orf49 as a regulator of SREBP processing and cholesterol metabolism. Nat Metab. 2020;2:487–98. 32694732 10.1038/s42255-020-0206-9 PMC7384252

[23] Price C, Gill S, Ho ZV, Davidson SM, Merkel E, McFarland JM, et al. Genome-Wide Interrogation of Human Cancers Identifies EGLN1 Dependency in Clear Cell Ovarian Cancers. Cancer Res. 2019;79:2564–79. 30898838 10.1158/0008-5472.CAN-18-2674 PMC6522283

[24] Niemi NM, Wilson GM, Overmyer KA, Vogtle FN, Myketin L, Lohman DC, et al. Pptc7 is an essential phosphatase for promoting mammalian mitochondrial metabolism and biogenesis. Nat Commun. 2019;10:3197. 31324765 10.1038/s41467-019-11047-6 PMC6642090

[25] Alsina D, Lytovchenko O, Schab A, Atanassov I, Schober FA, Jiang M, et al. FBXL4 deficiency increases mitochondrial removal by autophagy. EMBO Mol Med. 2020;12:e11659. 32525278 10.15252/emmm.201911659 PMC7338799

[26] Wilhelm LP, Zapata-Munoz J, Villarejo-Zori B, Pellegrin S, Freire CM, Toye AM, et al. BNIP3L/NIX regulates both mitophagy and pexophagy. EMBO J. 2022;41:e111115. 36215693 10.15252/embj.2022111115 PMC9753467

[27] Dunn WA Jr, Cregg JM, Kiel JA, van der Klei IJ, Oku M, Sakai Y, et al. Pexophagy: the selective autophagy of peroxisomes. Autophagy. 2005;1:75–83. 16874024 10.4161/auto.1.2.1737

[28] Iwashita H, Torii S, Nagahora N, Ishiyama M, Shioji K, Sasamoto K, et al. Live Cell Imaging of Mitochondrial Autophagy with a Novel Fluorescent Small Molecule. ACS Chem Biol. 2017;12:2546–51. 28925688 10.1021/acschembio.7b00647

[29] Niemi NM, Serrano LR, Muehlbauer LK, Balnis CE, Wei L, Smith AJ, et al. PPTC7 maintains mitochondrial protein content by suppressing receptor-mediated mitophagy. Nat Commun. 2023;14:6431. 37833277 10.1038/s41467-023-42069-w PMC10575892

[30] Sun Y, Cao Y, Wan H, Memetimin A, Cao Y, Li L, et al. A mitophagy sensor PPTC7 controls BNIP3 and NIX degradation to regulate mitochondrial mass. Mol Cell. 2024;84:327–44.e9. 38151018 10.1016/j.molcel.2023.11.038

[31] Wang Q The study of CG12091 on neuronal homeostasis maintenance in Drosophila. Zhejiang University; 2019.

