# Supplementary material for: PPTC7 acts as an essential co-factor of the SCFFBXL4 ubiquitin ligase complex to restrict BNIP3/3L-dependent mitophagy
Source: Cell Death Dis. 2025 Mar 1;16(1):145. doi: 10.1038/s41419-025-07463-w (PMC11873123; doi:10.1038/s41419-025-07463-w)
Supplement: Supplementary file 1 — Supplementary Figures [file 41419_2025_7463_MOESM1_ESM.pdf]

Supplementary Materials for

PPTC7 Acts as an Essential Co-factor of the SCF^{FBXL4} ubiquitin ligase complex to Restrict BNIP3/BNIP3L-dependent Mitophagy

Figures. S1 to S3

Other Supplementary Materials for this manuscript include the following:

Table S1 to S3

Supplementary Table 1. Sequence information.

Supplementary Table 2. Antibody information.

Supplementary Table 3. Cell cultures, chemicals, and kits.

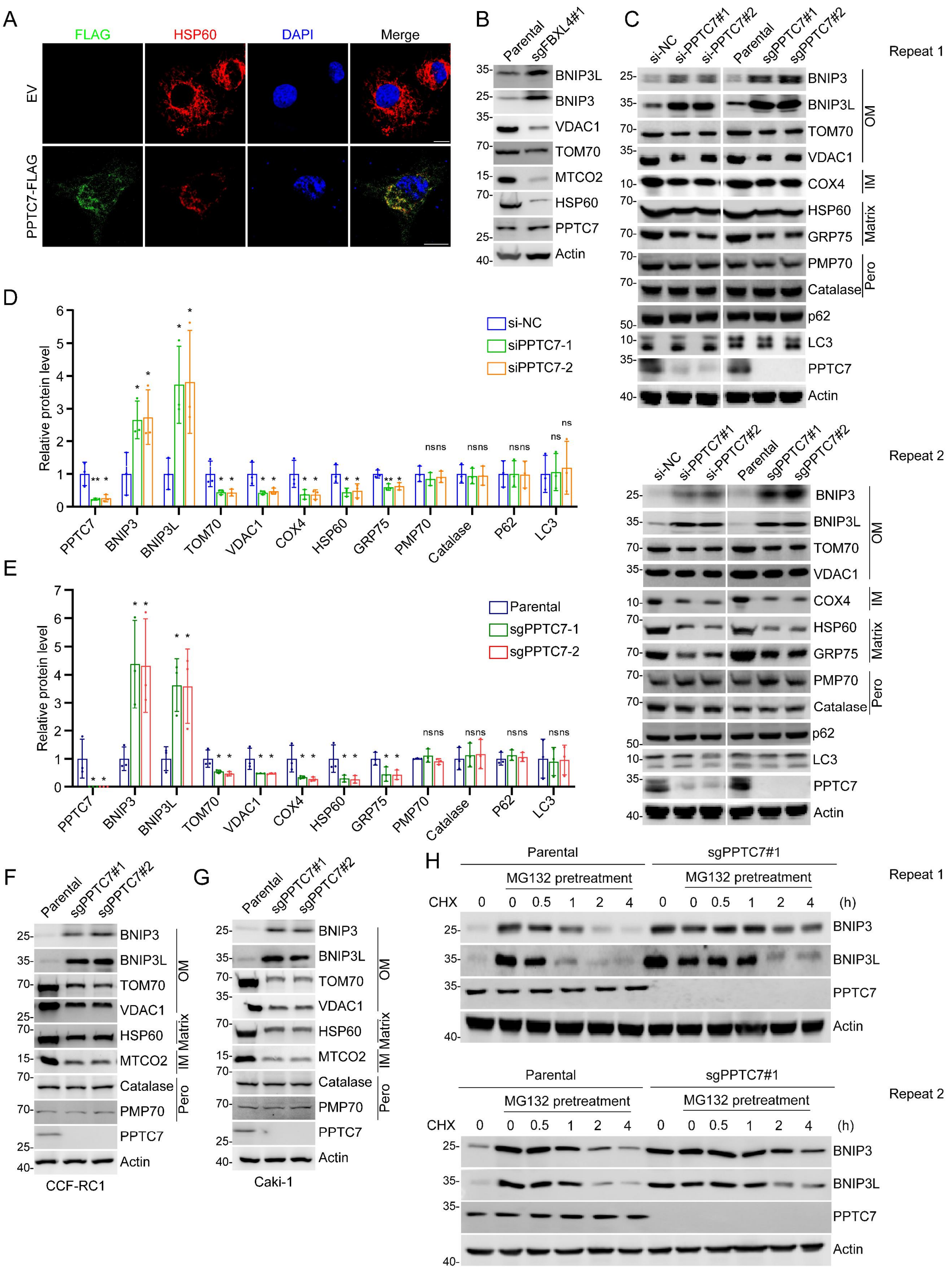

Supplementary Figure 1. PPTC7 controls BNIP3/3L protein stability in a protein phosphatase activity-independent manner (related to Figure 1, 2).

(A) Representative IF images from HeLa cells stably overexpressing EV, or PPTC7-FLAG, stained with FLAG, HSP60, and DAPI. Scale bar, 10 μm.

(B) WB analysis of the indicated proteins in parental and FBXL4 KO HeLa cells.

(C-E) WB analysis of the indicated proteins in HeLa cells transfected with either negative control

siRNAs (siNC) or PPTC7-specific siRNAs, as well as from parental and PPTC7 KO HeLa cells generated via LentiCRISPRv2. The indicated mitochondrial and cytoplasmic proteins were analyzed. Results represent three independent experiments. Two additional replicates of Fig. 2A are shown in (C), and quantitative analysis of three replicates is shown in (D, E). Data were shown as means ± SD. (n = 3).

(F, G) WB analysis of the indicated proteins in parental and PPTC7-KO CCF-RC1 (F) or Caki-1 (G) cells.

(H) WB analysis of BNIP3 and BNIP3L protein levels in parental and PPTC7 KO HeLa cells pretreated with DMSO or MG132 (20 μM, 5 h) before treatment with cycloheximide (CHX, 50 μg/mL). Samples were harvested at the indicated time points. Two additional replicates of Fig. 2H are shown.

P values are calculated by the Two-way ANOVA test in (D, E). $^*P < 0.05$; $^{**}P < 0.01$; n.s. non-significant.

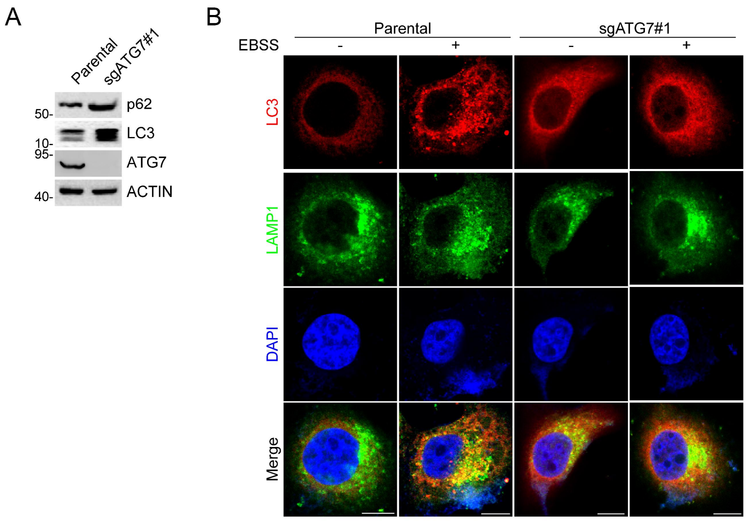

Supplementary Figure 2. ATG7 KO causes autophagic dysfunction, leading to the accumulation of p62 and LC3B (related to Figure 3).

(A) WB analysis of the indicated proteins in parental and ATG7 KO HeLa cells.

(B) Representative IF images from parental and ATG7 KO HeLa cells treated with (+) or without (-) EBSS media for 12 h, stained with LC3B, LAMP1, and DAPI. Scale bar, 10 μm.

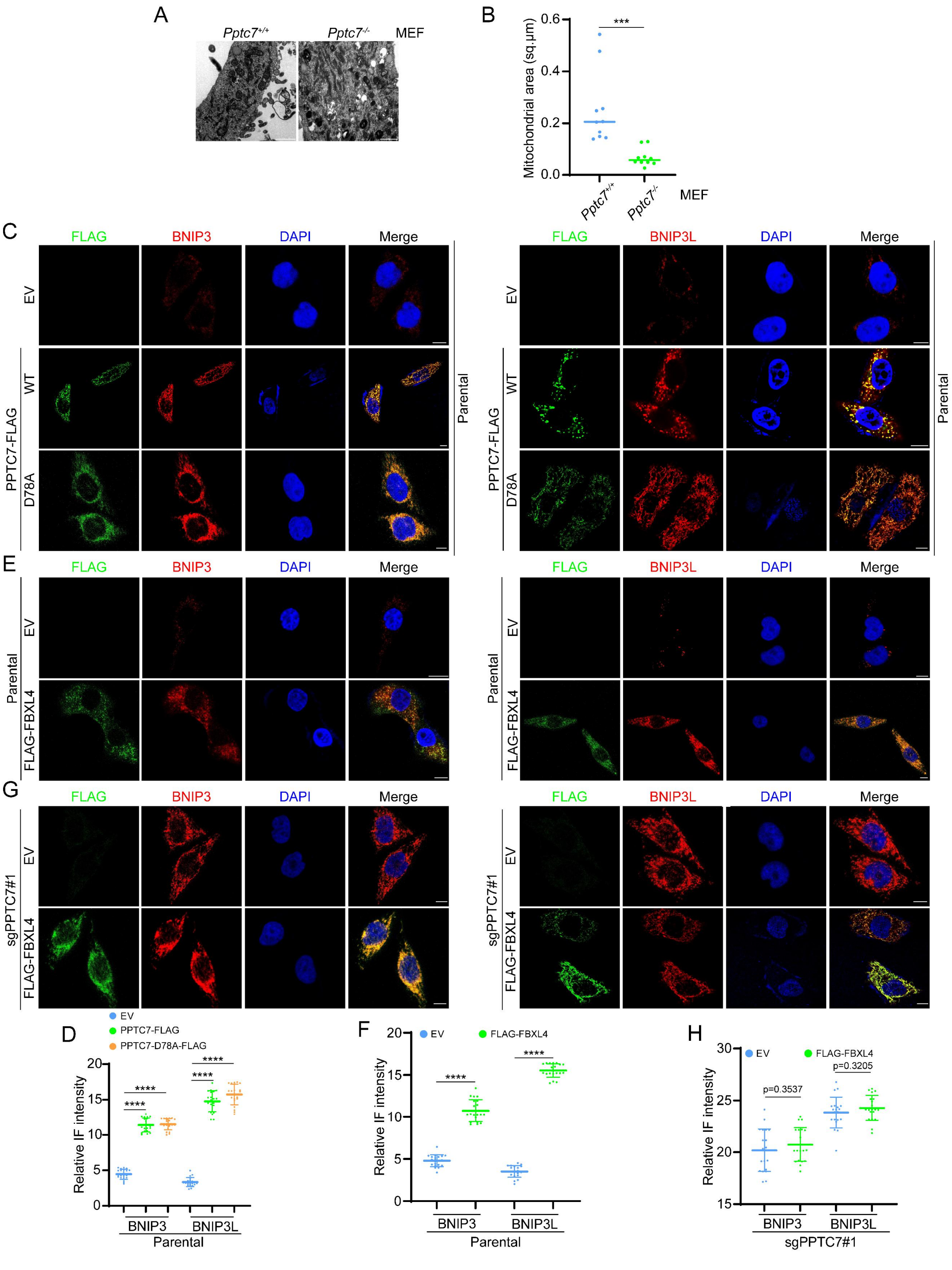

Supplementary Figure 3. PPTC7 is an essential co-factor of SCF^{FBXL4} E3 ubiquitin ligase complex (related to Figure 4, 5).

(A, B) *Pptc7*$^{+/+}$ and *Pptc7*$^{-/-}$MEFs were examined by transmission electron microscopy for mitophagic vacuoles. Scale bar, 1 μm. The statistical analysis of the representative mitochondrial area is shown in (B). Data were shown as means ± SD (n = 10 cells per group).

(C, D) Representative IF images from HeLa cells overexpressing the indicated plasmids, stained with BNIP3 (or BNIP3L), HSP60, and DAPI. Scale bar, 10 μm. The relative intensity of HSP60, BNIP3, and BNIP3L was quantified and shown in (D). Data were shown as means ± SD (n = 20 cells per group).

(E, F) Representative IF images from parental HeLa cells overexpressing the indicated plasmids, stained with BNIP3 (or BNIP3L), HSP60, and DAPI. Scale bar, 10 μm. The relative intensity of HSP60, BNIP3, and BNIP3L was quantified and shown in (F). Data were shown as means ± SD (n = 20 cells per group).

(G, H) Representative IF images from PPTC7 KO HeLa cells stably overexpressing EV or FLAG-FBXL4, stained with BNIP3 (or BNIP3L), HSP60, and DAPI. Scale bar, 10 μm. The relative intensity of HSP60, BNIP3, and BNIP3L were quantified and shown in (H). Data were shown as means ± SD (n = 20 cells per group).

P values are calculated by the One-way ANOVA test in (B) and the Two-way ANOVA test in (D, F, H). ***$P < 0.001$; ****$P < 0.0001$.
