# Supplementary material for: PPTC7 acts as an essential co-factor of the SCFFBXL4 ubiquitin ligase complex to restrict BNIP3/3L-dependent mitophagy
Source: Cell Death Dis. 2025 Mar 1;16(1):145. doi: 10.1038/s41419-025-07463-w (PMC11873123; doi:10.1038/s41419-025-07463-w)

Figure 1C

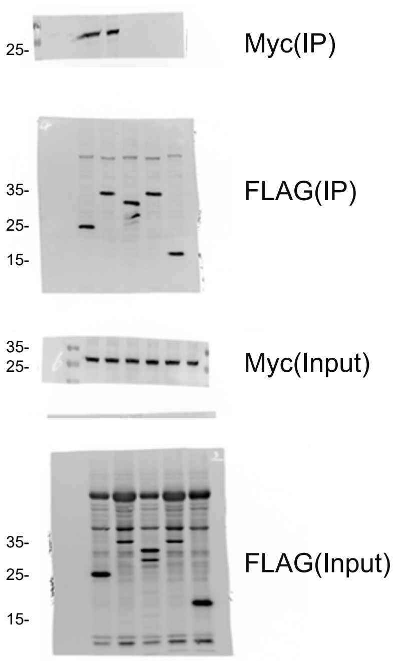

Figure 1D

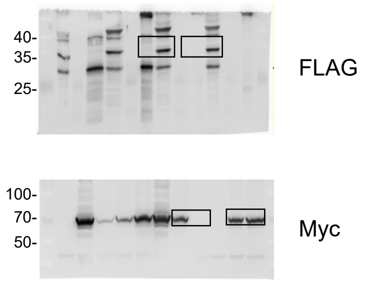

Figure 1E

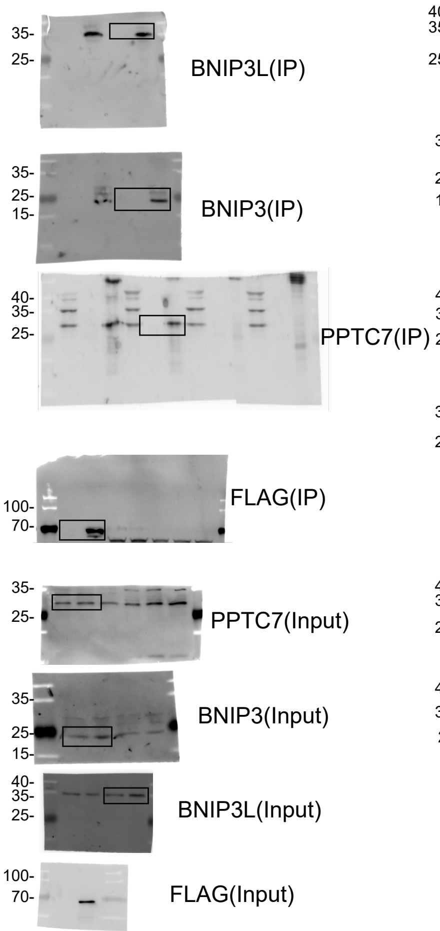

Figure 1F

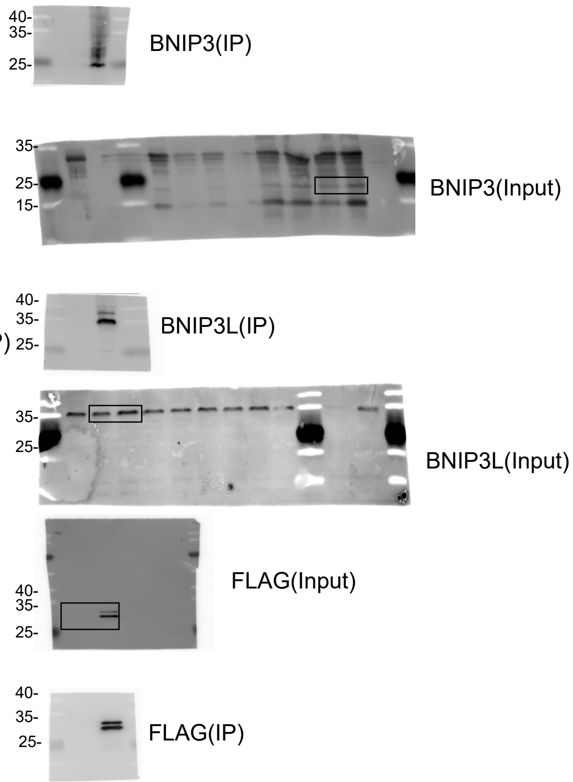

Figure 1G

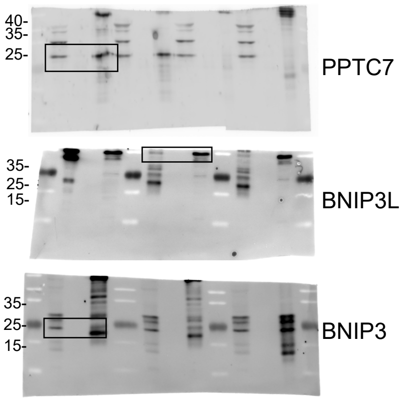

Figure 1H

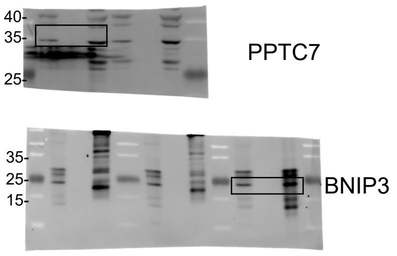

Figure 1I

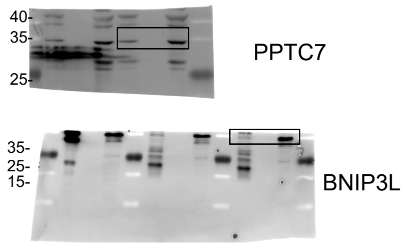

Figure 1J

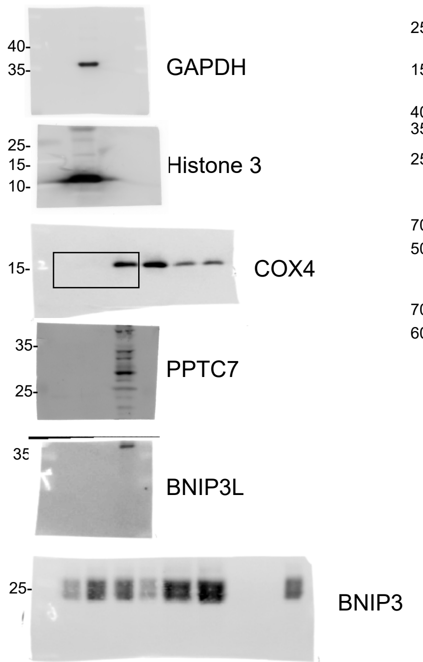

Figure 1K

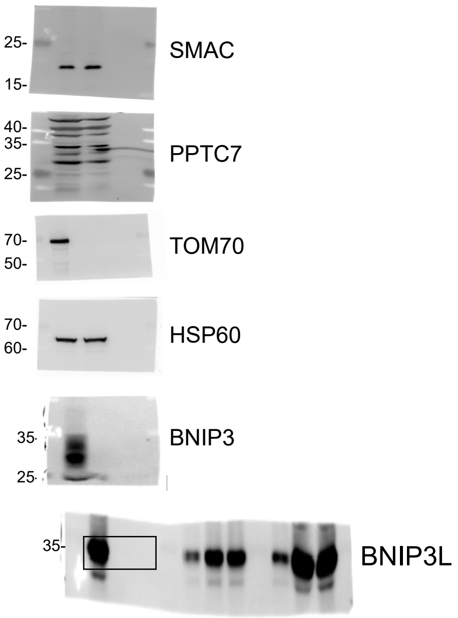

Figure 2A

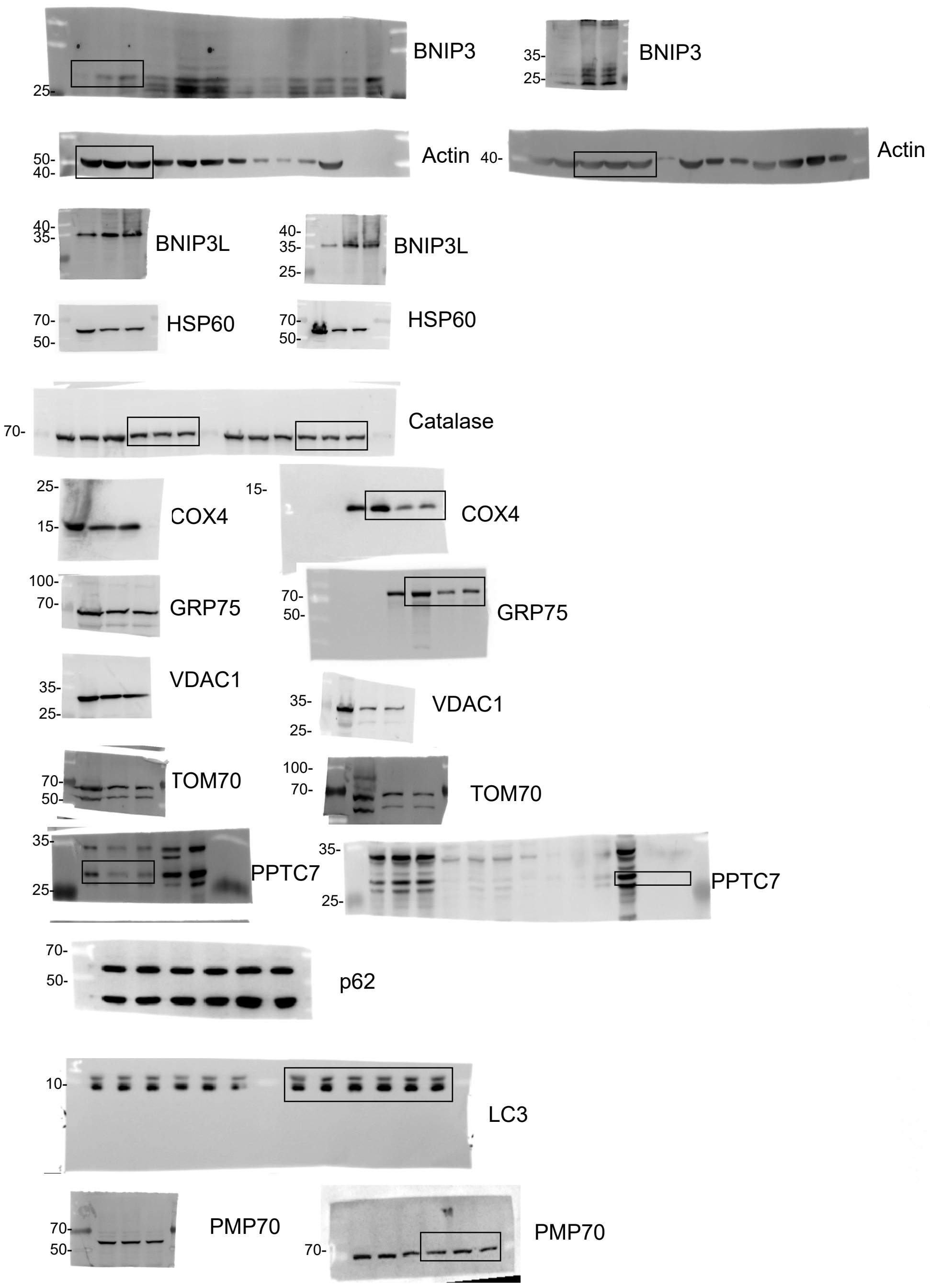

Figure 2C

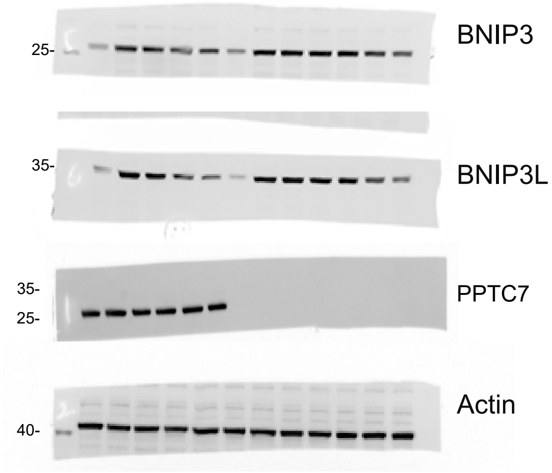

Figure 2H

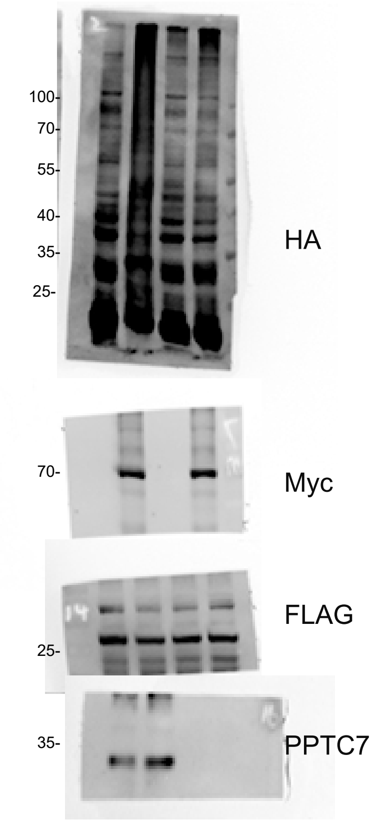

Figure 2I

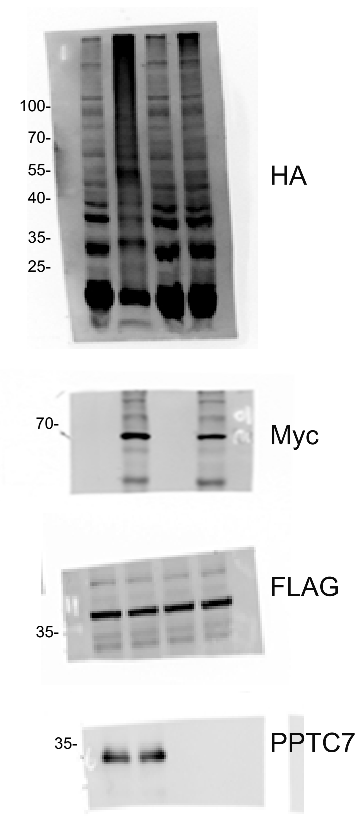

Figure 2J

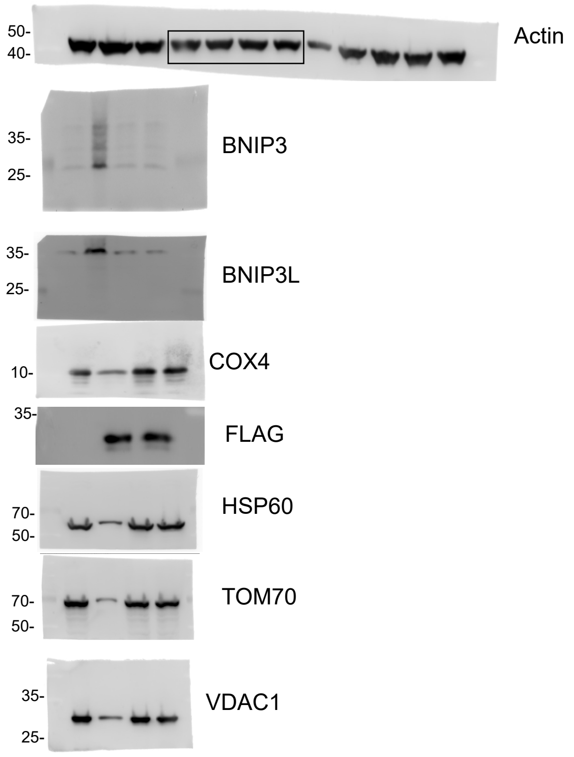

Figure 2K

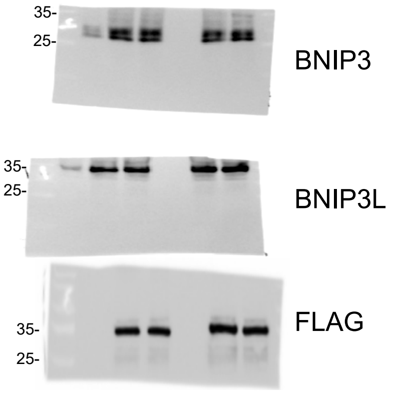

Figure 2L, M

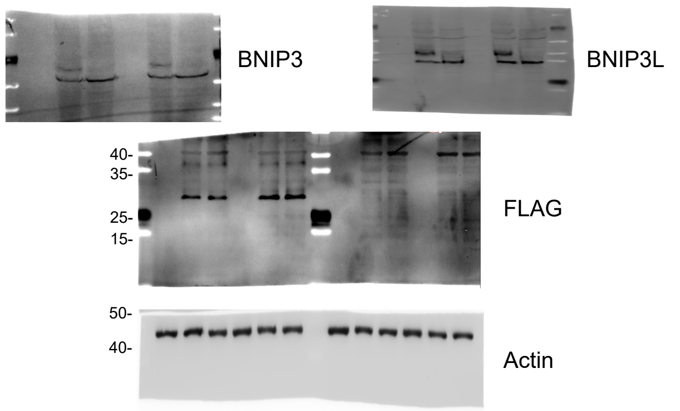

Figure 3A

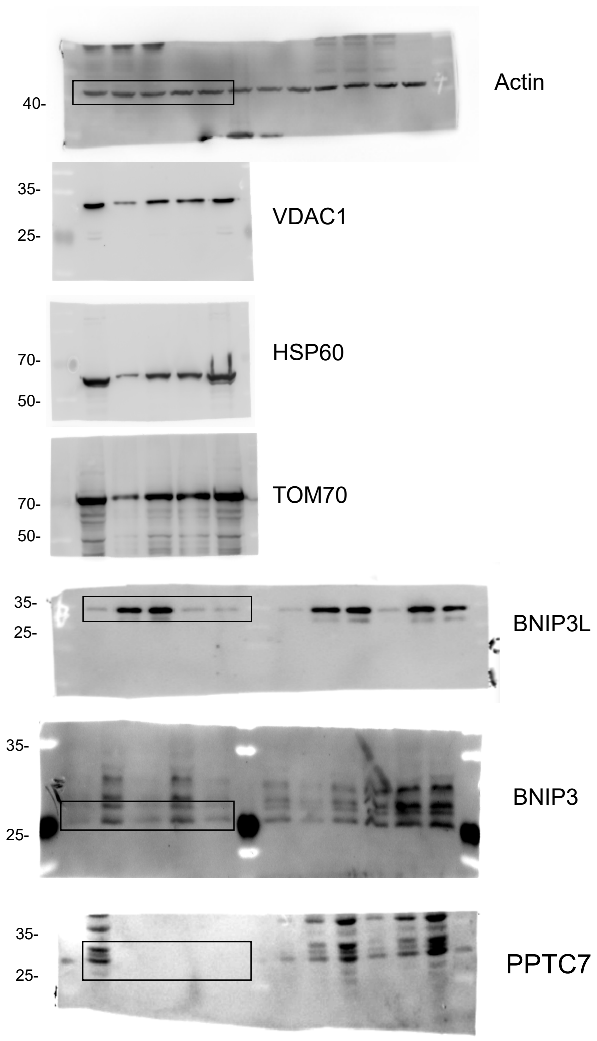

Figure 3B

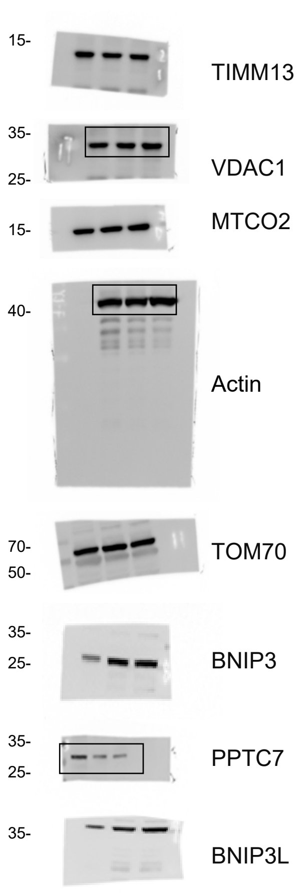

Figure 4B

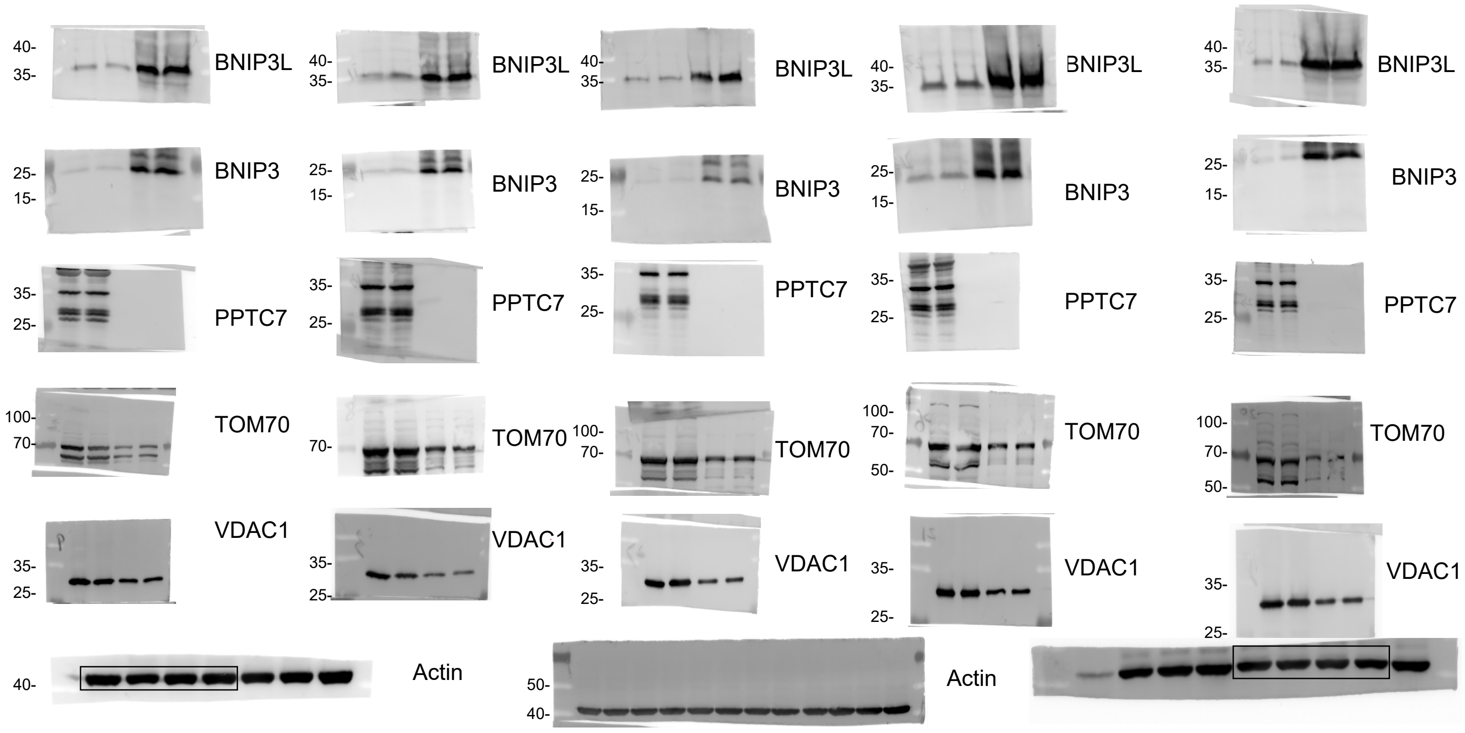

Figure 4C

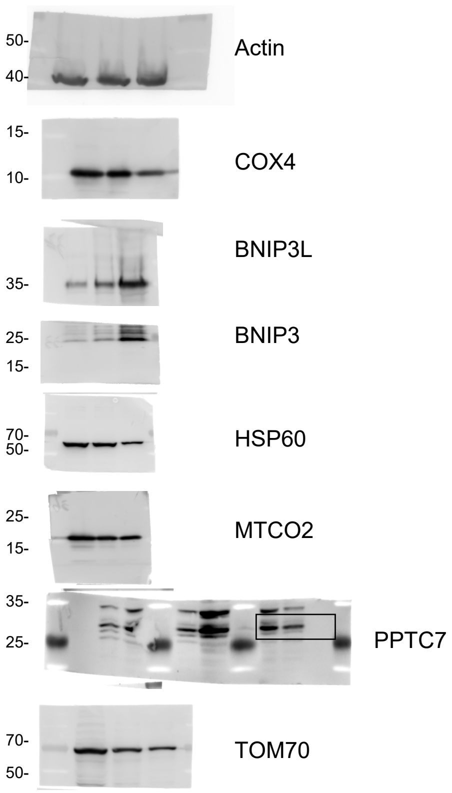

Figure 4H

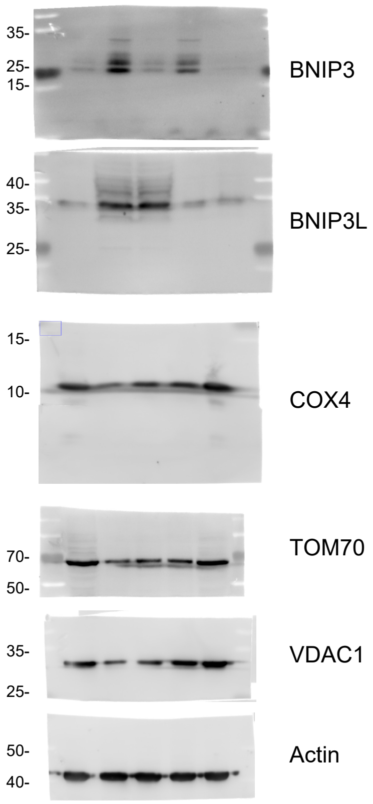

Figure 5A

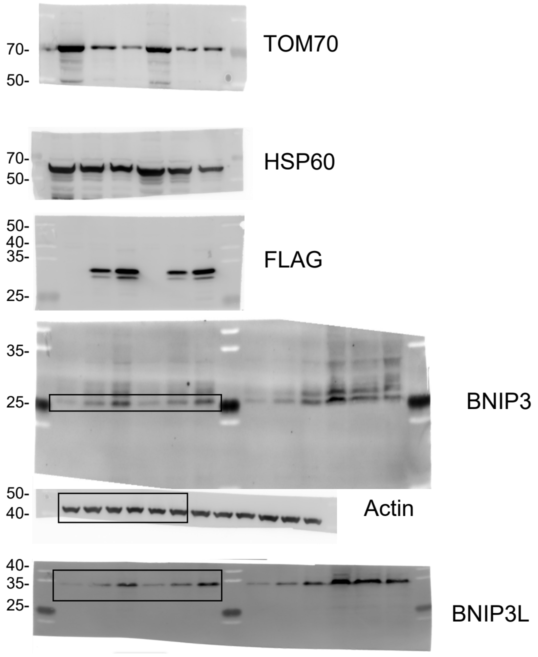

Figure 5C

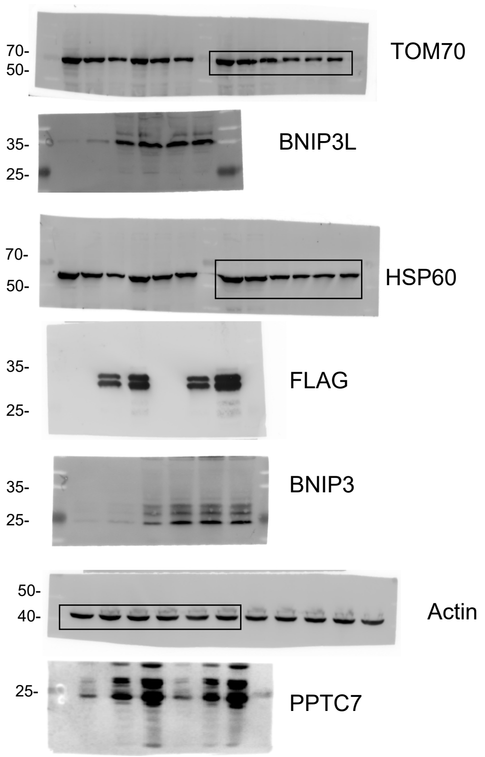

Figure 5F

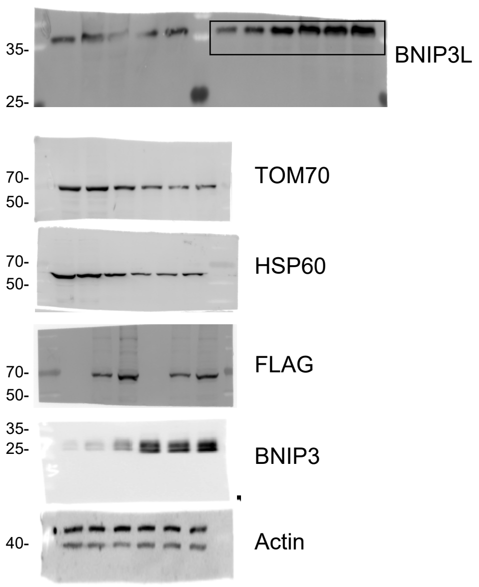

Figure 5G, I

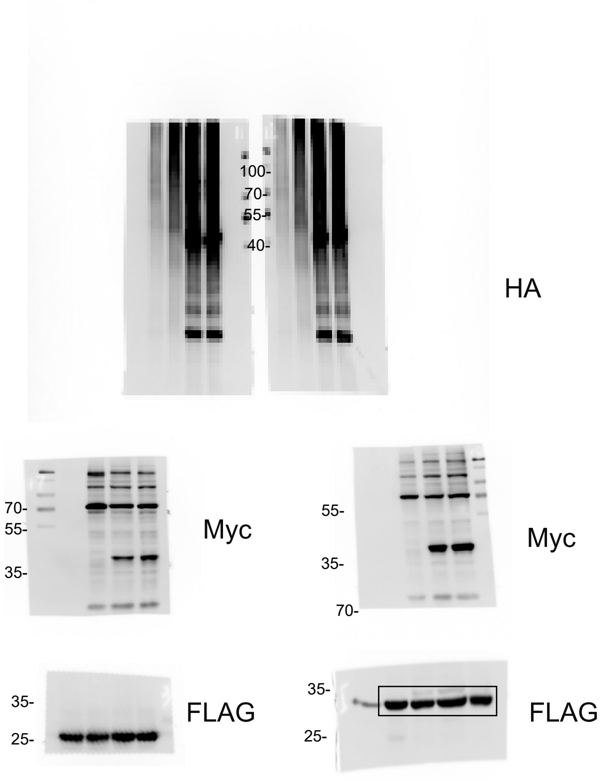

Figure 5H, J

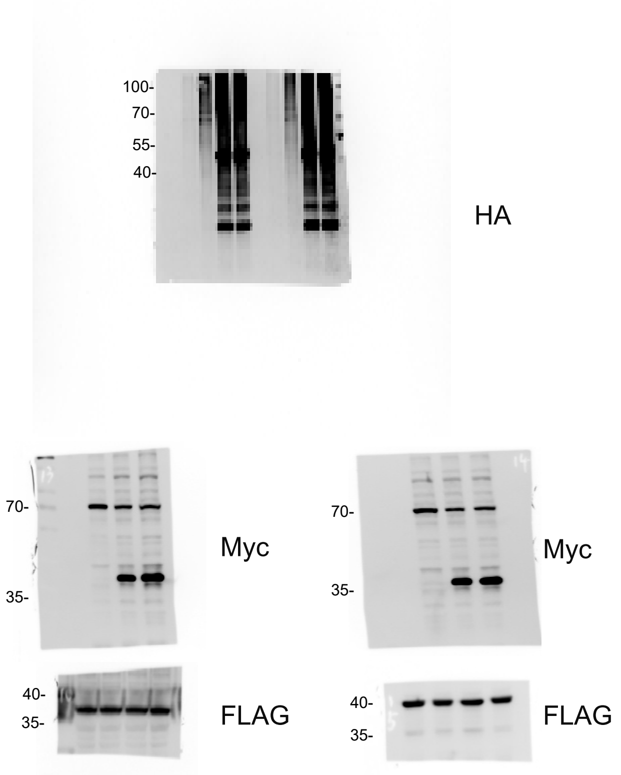

Figure S1B

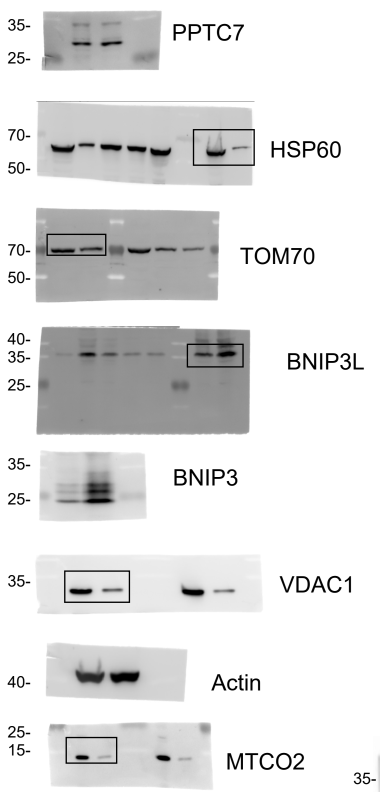

Figure S1C

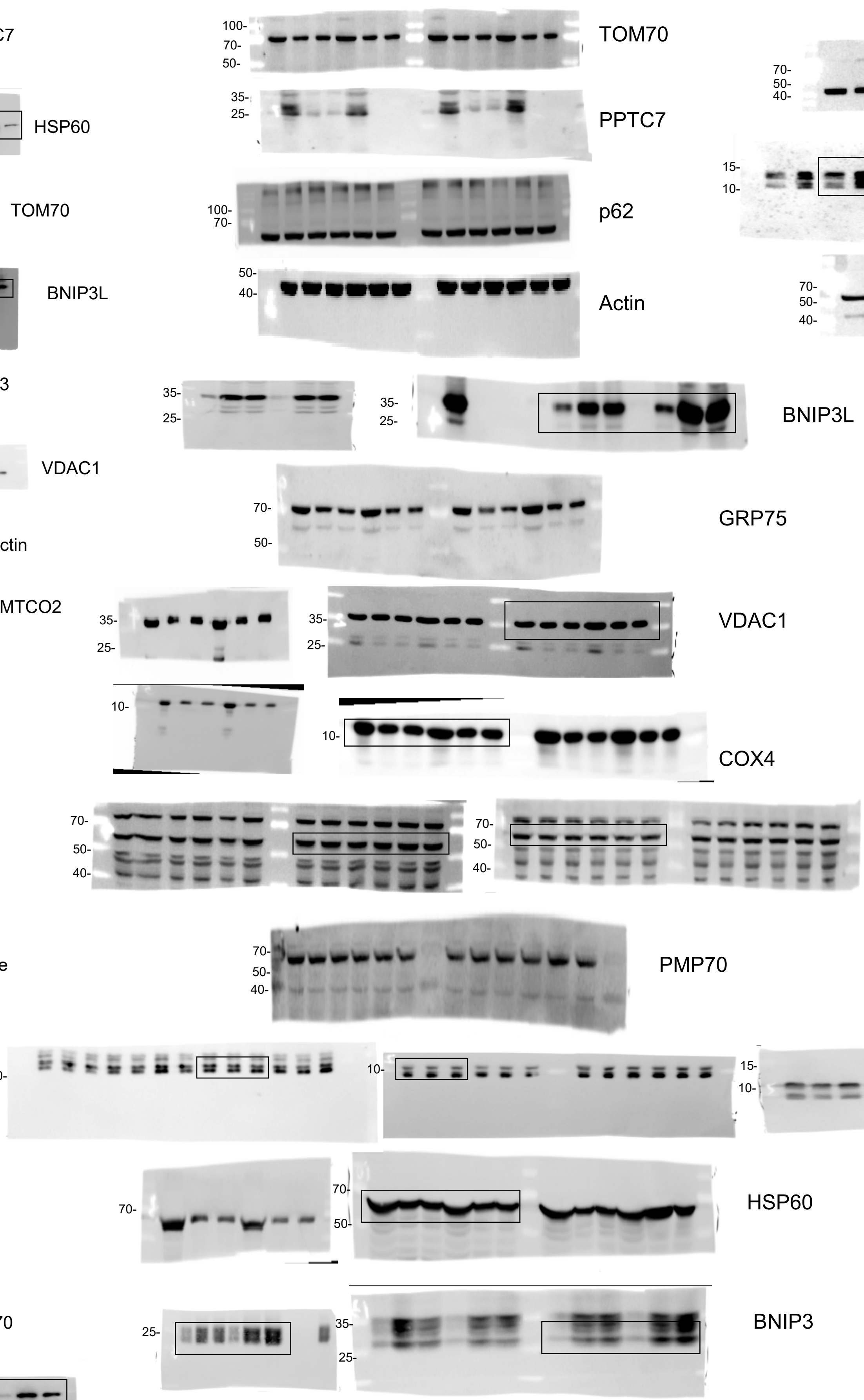

Figure S2A

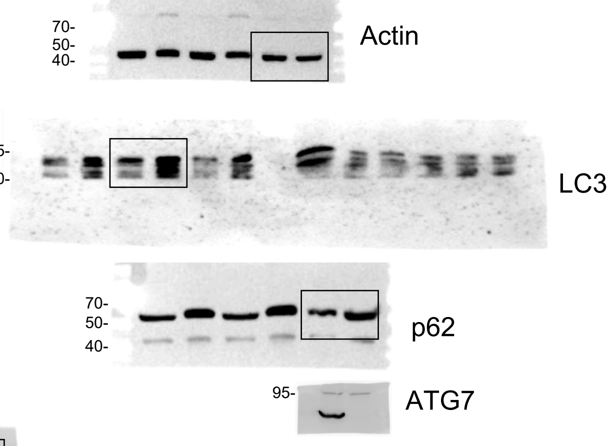

Figure S1F, G

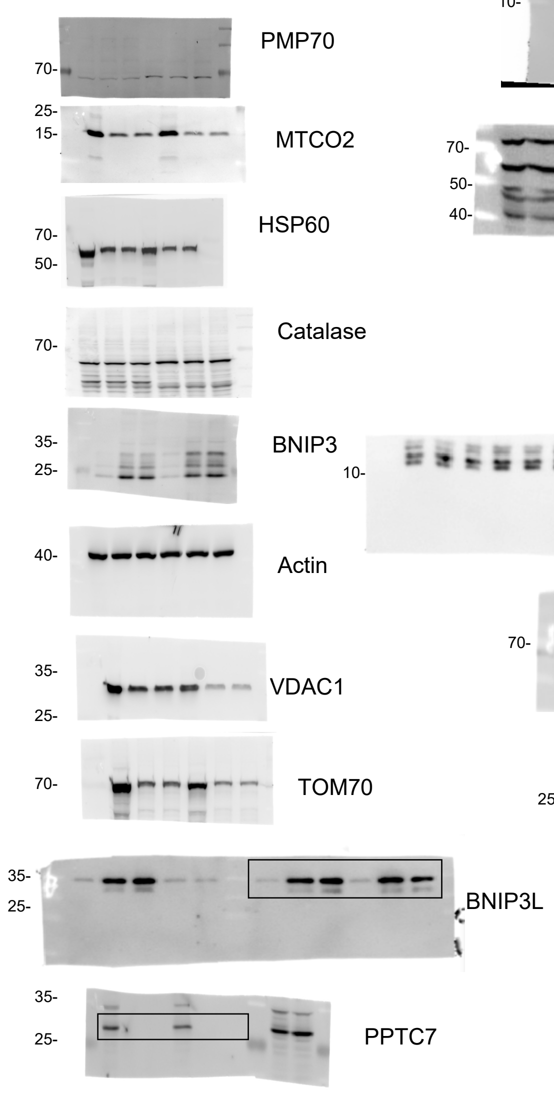

Figure S1H

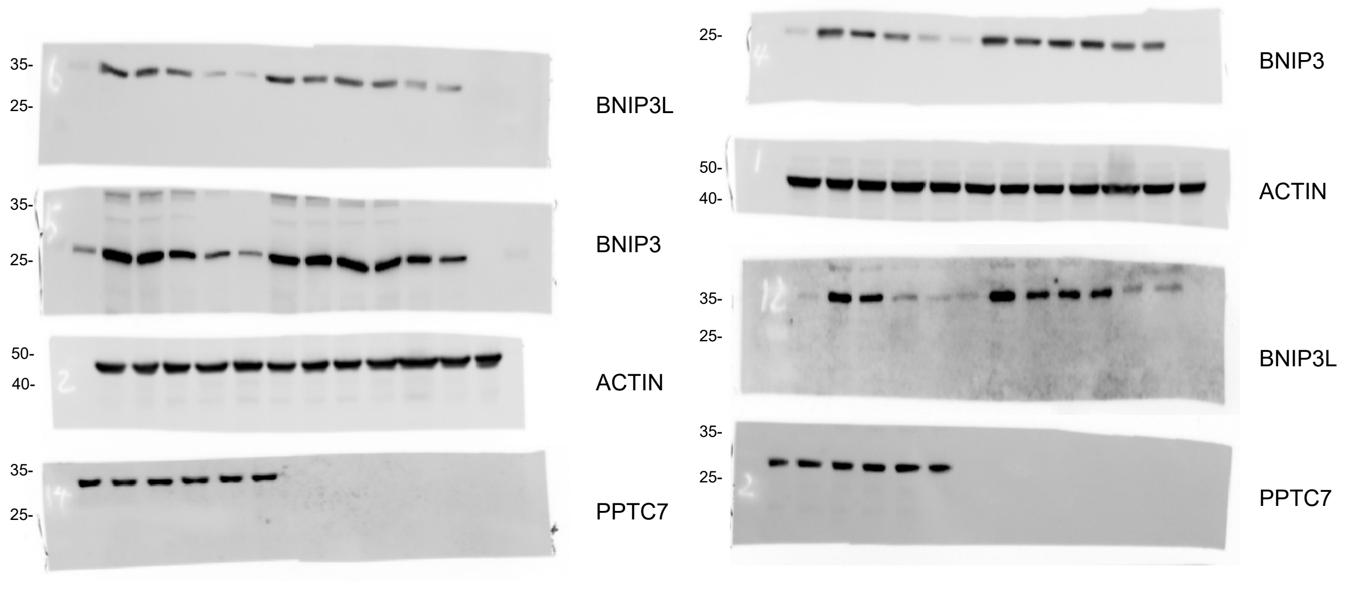

Supplement: Supplementary file 3 — Original Data [file 41419_2025_7463_MOESM3_ESM.pdf]
